# Simultaneous emulation of synaptic and intrinsic plasticity using a memristive synapse

**DOI:** 10.1038/s41467-022-30432-2

**Published:** 2022-05-19

**Authors:** Sang Hyun Sung, Tae Jin Kim, Hyera Shin, Tae Hong Im, Keon Jae Lee

**Affiliations:** 1 Department of Materials Science and Engineering, Korea Advanced Institute of Science and Technology (KAIST), 291 Daehak-ro, Yuseong-gu, Daejeon 34141 Republic of Korea; 2 Present Address: Memory Division, Samsung Electronics Co. Ltd., Pyeongtaek-si, Gyeonggi-do South Korea

**Keywords:** Electrical and electronic engineering, Electronic devices

## Abstract

Neuromorphic computing targets the hardware embodiment of neural network, and device implementation of individual neuron and synapse has attracted considerable attention. The emulation of synaptic plasticity has shown promising results after the advent of memristors. However, neuronal intrinsic plasticity, which involves in learning process through interactions with synaptic plasticity, has been rarely demonstrated. Synaptic and intrinsic plasticity occur concomitantly in learning process, suggesting the need of the simultaneous implementation. Here, we report a neurosynaptic device that mimics synaptic and intrinsic plasticity concomitantly in a single cell. Threshold switch and phase change memory are merged in threshold switch-phase change memory device. Neuronal intrinsic plasticity is demonstrated based on bottom threshold switch layer, which resembles the modulation of firing frequency in biological neuron. Synaptic plasticity is also introduced through the nonvolatile switching of top phase change layer. Intrinsic and synaptic plasticity are simultaneously emulated in a single cell to establish the positive feedback between them. A positive feedback learning loop which mimics the retraining process in biological system is implemented in threshold switch-phase change memory array for accelerated training.

## Abstract (web summary)

Synaptic plasticity and neuronal intrinsic plasticity are both involved in the learning process of hardware artificial neural network. Here, Lee et al. integrate a threshold switch and a phase change memory in a single device, which emulates biological synaptic and intrinsic plasticity simultaneously.

## Introduction

The intellectual capabilities of the human brain such as learning and memory emerge from the complex network of nearly a hundred billion neurons interconnected with synapses. A neuron combines the pre-synaptic input stimulus to fire electrical impulses, while a synapse connects adjacent neurons to transmit the signals throughout the network. Depending on the previous stimulus experiences, the function of neurons and synapses can be modified to reorganize the neural pathways. Synaptic plasticity, the ability of a synapse to adaptively change the connection strength, is well known for its contribution to learning and memorizing. Recently, numerous cellular and molecular studies reported that neurons not only participate in information processing, but also promote memory formation through intrinsic plasticity, which modulates neuronal excitability^1–3^. The synaptic plasticity and neuronal intrinsic plasticity occur concomitantly in all major forms of learning, allowing the brain to perform intelligent tasks and probabilistic processing with high efficiency^4,5^.

Inspired by the cognitive human brain, neuromorphic computing targets hardware embodiment of the biological neural network for the realization of artificial intelligence (AI)^6–8^. Device implementations of individual neurons and synapses have been widely investigated due to their excellent energy efficiency and scalability compared to CMOS-based approaches^9^. The development of artificial synapses has been greatly accelerated by the advent of memristor, which shows hysteretic resistance switching characteristics^10,11^. Both short- and long-term synaptic plasticity have been successfully emulated by nonvolatile memristors, owing to the strong resemblance of resistive switching behavior to synaptic plasticity^12–15^. Artificial neurons have also been demonstrated using volatile memristors, emulating neuronal models ranging from a bioplausible integrate-and-fire model to a biophysical Hodgkin-Huxley (HH) model^16–18^.

The integration of artificial neurons and synapses is essential for the development of neuromorphic intelligent computers with high-level cognitive functions^19^. Recently, memristive neural networks capable of pattern recognition and simple decision-making have been reported, showing superior performance over the conventional von Neumann architecture^20–24^. However, very few studies have demonstrated the emulation of intrinsic plasticity in an artificial neuron despite of important role in learning and memorizing^25,26^. In addition, the synergistic interaction between intrinsic and synaptic plasticity should be involved in various forms of learning such as classical conditioning, spatial learning, and retraining^4^. Although there have been several reports demonstrate the volatile and nonvolatile switching in a single device, these researches showed transition from volatile to nonvolatile switching, rather than a coexistence of both switching mechanism with neurosynaptic interactions^27–29^. The implementation of neuronal excitability and synaptic-weight change in a single device should be provided for the concomitant solution of neuroplasticity in brain-inspired cognitive AI.

Here, we report a synaptic device that mimics synaptic and intrinsic plasticity in a single unit cell for the interactive concomitance. An Ag-doped SiO_2_ TS and Ge_2_Sb_2_Te_5_ (GST)-based PCM are merged in a stack unit-cell of threshold switch-phase change memory (TS-PCM) to emulate an intrinsic and synaptic plasticity simultaneously. The nonvolatile phase transition of PCM layer is induced by Joule heating of the volatile Ag conductive filament (CF) in TS layer, showing high similarity to the synaptic weight modulation by neuron firing in a biological neural network. Due to the fully simultaneous nonvolatile and volatile resistive switching, both synaptic plasticity and neuronal intrinsic plasticity are emulated in a single cell of TS-PCM. In addition, a positive feedback learning loop is established based on the synergistic interaction of concomitant neuroplasticity. Finally, memorization and retraining of 4 × 4 patterns are successfully implemented by adopting the concomitant plasticity and feedback learning loop of TS-PCM.

## Results

### TS-PCM device with neuron-synapse pair structure

**Fig. 1:**
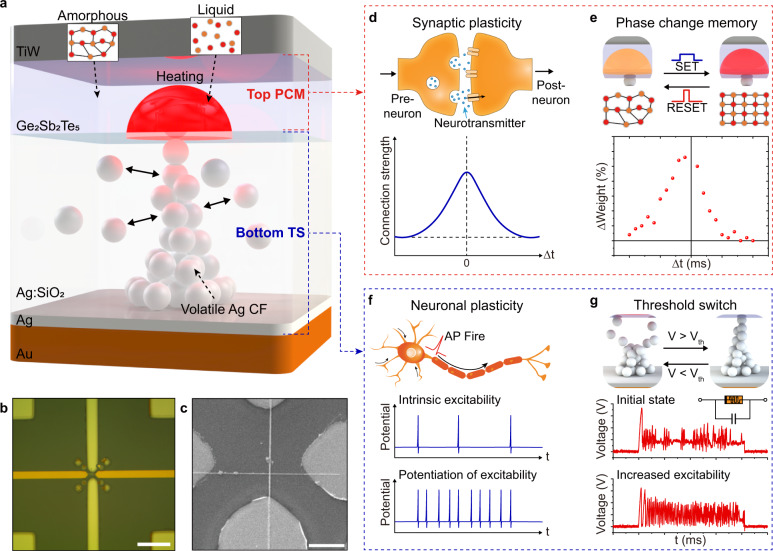
Structure of TS-PCM composed of volatile TS and nonvolatile PCM layers. **a** Schematic diagram of TS-PCM composed of volatile TS and nonvolatile PCM layer. The phase transition of the top PCM layer is regulated by the Ag filament formation in the bottom TS layer. **b** Optical microscope image of fabricated TS-PCM cell. Scale bar, 10 μm. **c** SEM image of TS-PCM showing the nanopatterned electrodes via EBL process. Scale bar, 5 μm. **d** Illustration of biological synapse with emission neurotransmitters (upper panel). A representative example of STDP is shown in the lower panel. **e** Schematic diagram of PCM with the phase transition process (upper panel). The lower panel demonstrates the emulation of STDP by TS-PCM. **f** Illustration of a biological neuron that integrates input signals into an AP spike (upper panel). The lower panel shows the neuronal intrinsic plasticity of neuron. **g** Schematic diagram of TS device presenting the formation and rupture of Ag filament (upper panel). The lower panel demonstrates the emulation of intrinsic plasticity by TS-PCM and a parallel capacitor.

TS-PCM is composed of a top non-volatile PCM layer and a bottom volatile TS layer without an intermediate electrode to implement memristive synapse with neuronal plasticity. As shown in Fig. 1a, we have developed a unique structure where the phase transition of PCM layer is regulated by the filament formation of TS layer, which mimics a biological neural network with the synaptic modulation of neuronal firing. The volatile TS layer is fabricated by co-sputtering of Ag and SiO_2_ targets after the deposition of an inert Au electrode to suppress the evolution of nonvolatile filaments^30^. A 1-nm-thick Ag layer is deposited at the Ag:SiO_2_/Au interface to decrease and stabilize the threshold voltage (*V*_*th*_) of CF formation (see Supplementary Fig. S1). The phase change material GST is directly deposited without metal interconnections, followed by the deposition of a TiW top electrode (see Methods and Supplementary Fig. S2 for fabrication details).

The pair structure of TS-PCM is designed to achieve coexistence of volatility and nonvolatility. A volatile Ag filament grows in the Ag-doped SiO_2_ TS layer by an electric field above *V*_*th*_, forming a contact with the GST/Ag:SiO_2_ interface. Due to the small contact area of CF, Joule heating is induced by the volatile Ag filament, resulting in a phase transition of the top GST film^31^. The phase of GST layer reversibly changes between amorphous and crystalline states depending on the applied pulse amplitude and width, leading to nonvolatile resistive switching^32^. After the filament-driven phase transition process, the Ag CF in the Ag:SiO_2_ layer spontaneously ruptures by the Gibbs-Thomson effect^33,34^, enabling the coexistence of volatility and nonvolatility in a single TS-PCM cell. Figure 1b presents an optical image of a fabricated TS-PCM cell with a simple crossbar structure. The device is patterned in 100 × 100 nm^2^ size via an e-beam lithography (EBL) process to improve the reproducibility of volatile CF formation in the TS layer (see Supplementary Figs. S3 and S11), as shown in the scanning electron microscope (SEM) image of Fig. 1c.

The nonvolatile resistive switching behavior of PCM layer is analogous to synaptic plasticity of a biological system, facilitating the implementation of an artificial synapse. As illustrated in the upper panel of Fig. 1d, a synapse is a gap between the dendrite of a post-neuron and the axon terminal of a pre-neuron, which transmits signals by the emission of neurotransmitters. Due to the synaptic plasticity, the strength of synaptic connection can be strengthened or weakened depending on the stimulation timing, frequency, and amplitude. The lower panel of Fig. 1d illustrates a representative example of synaptic plasticity, called spike-timing-dependent plasticity (STDP), where the connection strength is enhanced depending on the time interval between pre-synaptic and post-synaptic spikes. As shown in the experimental results of Fig. 1e, the PCM layer in the TS-PCM device can emulate synaptic behaviors due to the hysteretic nonvolatile resistive switching of GST film. Figure 1e demonstrates that the GST-based PCM can emulate STDP, which is essential for the memory and learning functions.

Neuronal plasticity, another element of a cognitive neural network, can be emulated by an Ag:SiO_2_-based TS device using volatile resistive switching behavior. The upper panel of Fig. 1f introduces neuronal excitation that integrates input stimulus to fire an action potential (AP). Due to the intrinsic plasticity, the firing rate of a neuron is modulated according to the stimulus history, as illustrated in the lower panel of Fig. 1f. Recent studies on volatile memristors such as TS and Mott devices have shown promising results in hardware realization of artificial neurons^35,36^. Figure 1g describes the volatile resistive switching in the bottom TS layer in which Ag CF grows and spontaneously ruptures depending on the electric bias. The lower panel of Fig. 1g demonstrates the voltage spiking behavior by current input, introducing the emulation of intrinsic plasticity by the TS-PCM circuit. TS-PCM is designed to implement an artificial synapse with neuronal intrinsic plasticity, which will be discussed in detail in a later section.

### Operation of volatile TS, nonvolatile PCM, and unified TS-PCM

**Fig. 2:**
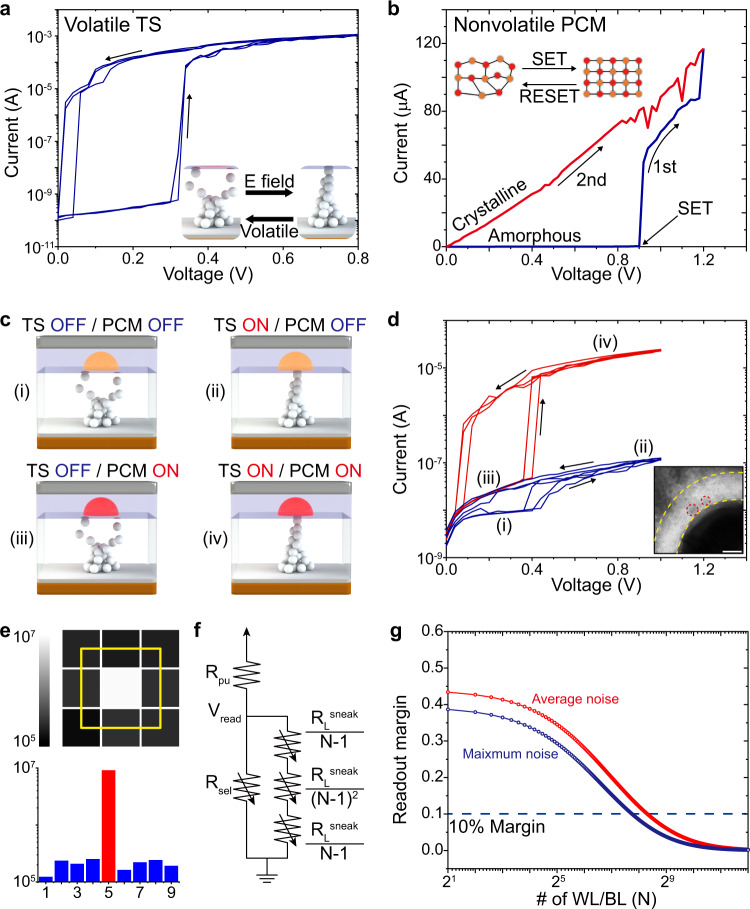
Electrical characteristics of volatile TS, nonvolatile PCM, and unified TS-PCM. **a** Current-voltage (I-V) curve of volatile TS device presenting threshold switching at 0.33 V and spontaneous reset. **b** I-V curve of PCM showing nonvolatile resistive switching by voltage sweep (blue). The second sweep (red) exhibits a high current value, indicating the nonvolatile phase transition of the GST film. **c** Schematic diagram of four stages in TS-PCM operation. Both TS and PCM layers are in the OFF state in stage (i) with high resistance. The TS layer is switched to the ON state in stage (ii) with relatively low resistance. The top PCM layer is switched to the ON state by Joule heating of the Ag filament in stage (iii), which shows high resistance due to the bottom TS layer. Both the TS and PCM layers are in the ON state in stage (iv), which shows the lowest resistance. **d** I-V curve of TS-PCM presenting the four stages illustrated in **c**. Inset shows a TEM image of the TS-PCM cell with Ag clusters in the SiO_2_ matrix. Scale bar, 10 nm. **e** Resistance-based color map (upper panel) and bar graph (lower panel) of worst-case scenario that verifies the random-access capability of TS-PCM. **f** Illustration of circuit model utilized in the OBPU method. For a sufficiently large *N*, R_L_^sneak^/(N-1)^2^ becomes negligible. **g** Calculated readout margin by OBPU method. The TS-PCM array can be scaled up to 316 × 316 for a 10% readout margin in average noise case (red curve). The maximum array size of TS-PCM is decreased to 214 × 214 in maximum noise case (blue curve).

Discrete cells of volatile TS and nonvolatile PCM are characterized for the unified operation of TS-PCM. The TS device, known as a diffusive memristor, is fabricated with a Pt/Ag:SiO_2_/Ag/Au structure to achieve stable volatile resistive switching performance^25^. As shown in Fig. 2a, the TS device in an initial high resistance state (HRS) is changed to a low resistance state (LRS) at *V*_*th*_ of 0.33 V with an on/off ratio over 5 × 10^5^. As illustrated in the inset images, the conductive Ag filament ruptures below *V*_*th*_, resulting in the transition to the HRS without an additional reset process. The high surface energy of Ag/SiO_2_ interface induces spontaneous rupture of the filament, leading to atomic clustering of Ag atoms in SiO_2_ host matrix^33^. In contrast, PCM exhibits nonvolatile resistive switching, as demonstrated in Fig. 2b. The as-deposited GST film is in an amorphous phase with high resistance of 1.11 × 10^4^ Ω, that changes to a crystalline phase by Joule heating during the voltage sweep. The operation current of PCM is dependent on the size of Joule heating element, which shows a dramatic decrease with the adoption of a conductive filament nanoheater, as reported in our previous paper^31^.

The TS-PCM device is fabricated by combining two distinctive volatile and nonvolatile memristive devices without intermediate electrodes between them. Because of the coexisting nonvolatile-volatile characteristics of TS-PCM, four different states are observed during the voltage-sweep operation, as shown in Fig. 2c, d. In the initial stage (i), the bottom TS and top PCM layers are in the OFF state, demonstrating high resistance over 3 × 10^7^ Ω. The bottom TS layer is switched to the ON state in stage (ii) by applying an electric field above *V*_*th*_, presenting relatively low resistance of 8 × 10^6^ Ω. The top PCM layer still remains in the OFF state in stage (ii), since the applied current during the 0-1 V voltage-sweep is insufficient to induce the phase transition of GST film. After the voltage sweep, TS-PCM is switched back to stage (i), presenting the volatility of the TS-PCM device. The PCM layer is changed to the ON state by application of a SET pulse (2 V, 200 ms), that leads to a transition to stage (iii). The TS-PCM in stage (iii) demonstrates high resistance of 9 × 10^6^ Ω, due to the TS layer being in the OFF state. A voltage bias above *V*_*th*_ induces the formation of Ag CF, which results in stage (iv) with low resistance of 4 × 10^4^ Ω. The bottom TS layer operates in a volatile manner regardless of the top PCM layer resistance, enabling the coexistence of volatile and nonvolatile resistive switching characteristics. Note that the coexistence of volatility and nonvolatility in TS-PCM is different from the volatile-nonvolatile transition behaviors reported elsewhere. In volatile-nonvolatile transition behavior, volatility is not maintained after the nonvolatile switching of the device. The power consumption level of TS-PCM has also been calculated in pulse measurement scheme (see Supplementary Fig. S14 for details). The set and reset power TS-PCM cell are 216 μW (129.6 nJ/bit) and 2.21 mW (0.33 nJ/bit), using 2 V amplitude and 600 μs width set pulse and 10 V amplitude and 50 ns width reset pulse, respectively. The reset current of TS-PCM is ~230 μA, which is lower than that of conventional PCM devices due to the filament confinement effect^37,38^. Note that the power efficiency of TS-PCM can be improved through the optimization of measurement setups and the scaling of cell size, owing to the low set and reset current values. The switching characteristics of TS-PCM can be modelled by the combination of phase change memory and threshold switch^39–42^. Using chalcogenide-based resistor function, Fermi-like smooth blending function, and filament-based resistance functions, the model of TS-PCM can be established (see Supplementary note 3)^42,43^.

The volatility of bottom TS layer prevents the sneak current problem that arises from the 2-terminal crossbar structure of TS-PCM^44^. Figure 2e demonstrates the random-access test results of a 3 × 3 TS-PCM array. The resistance-based color map and corresponding cell resistance graph of worst-case scenario indicate that the TS-PCM array operates in a random-access manner without the sneak current problem. The maximum array size of TS-PCM is calculated using the one-bit-line pull-up (OBPU) method^45–47^. The OBPU scheme utilizes a simplified circuit model to represent an *N* × *N* crossbar array, as shown in Fig. 2f. The normalized readout margin is given as follows:

1 $$\frac{\triangle V}{{V}_{{pull}-{up}}}=\frac{{R}_{{pull}-{up}}}{\left[{R}_{{LRS}}\parallel {R}_{{Sneak}}\right]+{R}_{{pull}-{up}}}-\frac{{R}_{{pull}-{up}}}{\left[{R}_{{HRS}}\parallel {R}_{{Sneak}}\right]+{R}_{{pull}-{up}}}$$

where *ΔV*, *V*_*pull-up*_, *R*_*pull-up*_, *R*_*LRS*_, *R*_*HRS*_, and *R*_*Sneak*_ are the readout voltage swing, pull-up voltage, pull-up resistance, LRS cell resistance, HRS cell resistance, and sneak path resistance, respectively. The calculation results in Fig. 2g indicate that the TS-PCM array can be scaled up to 316 × 316 for a 10% readout margin, verifying the random-access capability of TS-PCM device. In addition, the effect of noise on the maximum array size can be calculated using the resistance values with largest errors (see Supplementary Fig. S15). The resistance values for maximum noise case are 9.69 × 10^4^ Ω, 7.78 × 10^5^ Ω, and 2.32 × 10^7^ Ω, for *R*_*LRS*_, *R*_*HRS*_, and *R*_*L*_^*Sneak*^, respectively. For the same readout margin of 10%, possible array size of TS-PCM is calculated as 214 × 214 which is far less than the average noise case. (see Supplementary note 1 for calculation details).

### Simultaneous emulation of spiking behavior and synaptic plasticity

**Fig. 3:**
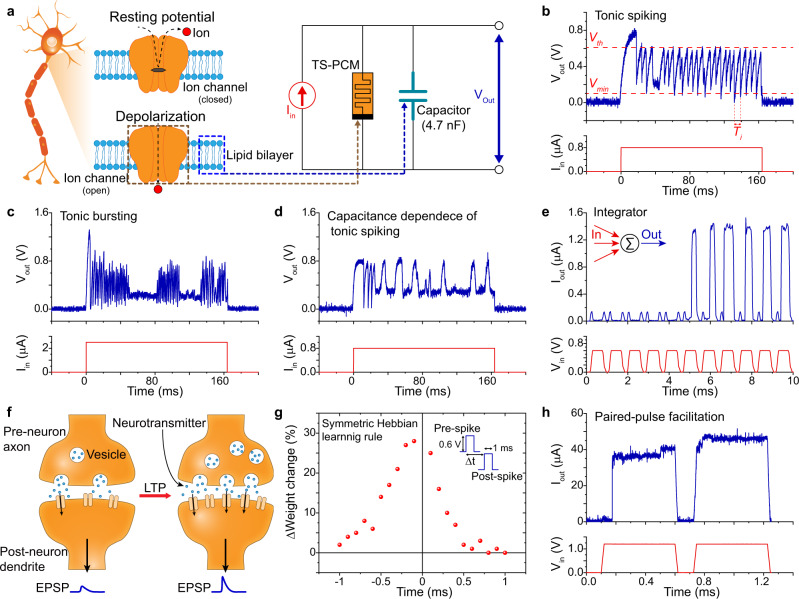
Emulation of spiking behavior and synaptic plasticity by TS-PCM. **a** Illustration of neuronal membrane structure (left) and corresponding circuit representation by TS-PCM (right). TS-PCM and the parallel capacitor are equivalent to a voltage-gated ion channel and lipid bilayer, respectively. **b** Tonic spiking behavior of TS-PCM using a 4.72 nF capacitor. The voltage spikes are generated by the charging and discharging of the capacitor with threshold switching of TS-PCM. *V*_*th*_ and *V*_*min*_ are measured to be 0.61 V and 0.1 V, respectively. *T*_*i*_ is a period of *i* th voltage spike from total n spikes, where average spike period $T=\sum {T}_{i}/n$. **c** Tonic bursting of TS-PCM showing rapid voltage spiking with quiescent periods. **d** Tonic spiking of TS-PCM with 5.61 nF capacitor. The firing frequency shows clear dependency on the capacitance. **e** Emulation of LIF behavior by TS-PCM. Consecutive input signals are integrated with continuous decay, which results in the generation of current spikes for the sixth input signal with a sufficiently short time interval. **f** Schematic diagram of synaptic plasticity showing before and after the LTP of synapse. Synaptic vesicles in the pre-neuron and dendritic receptors in the post-neuron increase after the LTP process, leading to enhanced connection strength and EPSP output. **g** Emulation of STDP using TS-PCM presenting a symmetric Hebbian learning rule. The time interval between pre-spike and post-spike determines the amount of synaptic weight change. Same pulse conditions are used for pre- and post-spike, which are 0.6 V amplitude and 1 ms pulse width. The device was reset between each measurement. **h** Short-term synaptic plasticity of TS-PCM. Paired-pulse facilitation is emulated based on the volatility of the bottom TS layer. Repetitive stimulations induce temporary increment of the filament radius, leading to an increase of current level for same voltage pulses.

The coexistence of volatile and nonvolatile resistive switching behaviors in TS-PCM enables the implementation of neuronal and synaptic behaviors in a single cell. Neuronal spiking behavior, a key element of information processing, has been emulated using the volatile characteristics of bottom TS layer in TS-PCM. Figure 3a describes the neuronal membrane in the resting potential state and depolarization state, with a corresponding circuit representation. Generation and propagation of AP spikes in the neuron are governed by the movement of charged ions such as Na^+^, K^+^, and Cl^−^ that create a certain potential difference across the lipid bilayer. The voltage-gated ion channels in the membrane open when pre-neuron neurotransmitters bind to post-neuron receptors, leading to depolarization of the neuronal membrane by the movement of ions. As shown in the right panel of Fig. 3a, the lipid bilayer and voltage-gated ion channel can be represented with simplified RC circuit with TS-PCM^16,48^. The bottom TS layer, parallel capacitor, and electrons are equivalent to the voltage-gated ion channel, lipid bilayer, and charged ions in a biological system, respectively. The capacitor separates charges and generates the potential difference similar to the lipid bilayer, while TS-PCM modulates current flow depending on Ag filament formation in the manner of the voltage-gated ion channel. It is noteworthy that the parallel capacitor in Fig. 3a is not essential for the spiking emulation of TS-PCM. Due to the inherent parasitic capacitance of the device, which is known to be proportional to the device area, TS-PCM is able to mimic the neuronal spiking behaviors without parallel capacitor units (see Supplementary Fig. S17). The parallel capacitor in Fig. 3a is adopted for the exact control of capacitance which provides comprehensive understanding of voltage spiking behaviors of TS-PCM.

Due to the functional similarity, TS-PCM can emulate various forms of neuronal spiking behavior like tonic spiking and bursting, as demonstrated in Fig. 3b–e^49–51^. Tonic spiking mode, a series of single spike firing, is known to be involved in working memory, fear extinction, and visual information processing. The emulation of tonic spiking mode is confirmed in Fig. 3b using TS-PCM and a parallel capacitor, showing repetitive and regular voltage spikes upon a current pulse input of 0.8 μA. On the other hand, tonic bursting mode, rapid firing with quiescent periods, governs low frequency stimulation and retrieval of previous learning^52–55^. Figure 3c indicates that tonic spiking behavior is changed to tonic bursting mode, presenting rapid firing with quiescent periods over 20 ms by a high input current of 2.5 μA. The voltage spiking behavior originates from the charging and discharging of capacitor in the parallel RC circuit. For tonic spiking mode, the voltage functions of the *n*th charging state (*V*_*n*_^*ch*^) and *n*th discharging state (*V*_*n*_^*dis*^) are given as follows:

2 $${V}_{n}^{{ch}}\left(t\right)={{I}_{{in}}R}_{H}\left\{1-{{{{{\rm{exp }}}}}}\left(-\frac{t-{t}_{n}^{{ch}}}{{\tau }_{{ch}}}\right)\right\}$$

3 $${V}_{n}^{{dis}}\left(t\right)=I{R}_{L}{{{{{\rm{exp }}}}}}\left(-\frac{t-{t}_{n}^{{dis}}-{\tau }_{{dis}}{{{{{\rm{ln}}}}}}\left({V}_{{th}}/I{R}_{L}\right)}{{\tau }_{{dis}}}\right)$$

where *I*_*in*_, *t*_*n*_^*ch*^, *t*_*n*_^*dis*^, *τ*_*ch*_, and *τ*_*dis*_ are the input current, charging time of the *n*th spike, discharging time of the *n*th spike, time constant of charging, and time constant of discharging, respectively. When *I*_*in*_*R*_*H*_ > *V*_*th*_ > 0 V, the capacitor voltage increases up to *V*_*th*_ according to the charging function of Eq. (2). The resistance of TS-PCM abruptly decreases from *R*_*H*_ to *R*_*L*_ after the TS switching above *V*_*th*_, leading to the discharging of parallel capacitor. Capacitor voltage decreases from *V*_*th*_ to 0 V following the discharging function of Eq. (3). The resistance of TS-PCM is switched back to *R*_*H*_ by the spontaneous reset of TS layer, that leads to charging of the capacitor. The charging and discharging of the capacitor iteratively occur by the repetitive threshold switching of TS-PCM, resulting in the voltage spiking behavior (see Supplementary note 2 for details).

The spiking voltage functions in Eqs. (2) and (3) are equivalent to the membrane voltage function in a single compartment model of a biological neuron that facilitates the implementation of neuronal functions by TS-PCM. For example, the capacitance of RC circuit determines the time constant *τ* *=* *RC*, modulating the speed of charging and discharging. The specific membrane capacitance (*C*_*m*_) of neuron has been reported as 1.0 μF/cm^2^ with a time constant of 100–200 ms^56^. The time constant of neuron depends on its surface area, affecting the propagation speed of AP through the membrane^57^. For the TS-PCM circuit with a 4.72 nF capacitor in tonic spiking mode, the time constant is measured to be 5.09 ms for *τ*_*ch*_ and 0.12 ms for *τ*_*dis*_, presenting a faster response time compared to a biological neuron (see Supplementary Fig. S12). In addition, the parallel capacitor regulates the firing frequency of TS-PCM tonic spiking. From Eqs. (2) and (3), spike period *T* can be calculated as follows:

4 $$T ={\tau }_{ch}{{{{{\rm{ln}}}}}}\left(\frac{{I{R}_{H}-V}_{{\min }}}{I{R}_{H}-{V}_{th}}\right)+{\tau }_{{dis}}{{{{{\rm{ln}}}}}}\left(\frac{{V}_{th}}{{V}_{{\min }}}\right)\\ =C\left[{R}_{H}{{{{{\rm{ln}}}}}}\left(\frac{{I{R}_{H}-V}_{{\min }}}{I{R}_{H}-{V}_{th}}\right)+{R}_{L}{{{{{\rm{ln}}}}}}\left(\frac{{V}_{th}}{{V}_{{\min }}}\right)\right]$$

Since the capacitance *C* is proportional to the time constant *τ*, the spike period *T* is proportional to the capacitance. As shown in Fig. 3d, it is confirmed that the average spike period is increased from 5.83 ms to 6.82 ms by increasing the capacitance from 4.72 nF to 5.61 nF. Furthermore, the charging and discharging characteristics facilitate the emulation of leaky integrate-and-fire (LIF) model in TS-PCM. The LIF model describes the generation of an AP spike as the integration of input signals with continuous decay. Due to the exponential decay of integrated potential, high frequency stimulation is required for the generation of an AP spike. Figure 3e demonstrate the emulation of LIF behavior using two consecutive input voltage pulses. Due to the low amplitude below *V*_*th*_ of the bottom TS layer, a single pulse input cannot generate a current spike of TS-PCM. For a sufficiently short time interval, two input signals are integrated above *V*_*th*_, firing a current spike, as shown in Fig. 3e. It should be noted that the *V*_*th*_ of TS-PCM in Fig. 3e is larger than the previous result shown in Fig. 2d. The threshold voltage of electrochemical metallization cells, like threshold voltage, depends on the measurement parameters such as amplitude, width, and delay time. Due to the short pulse width of 700 μs, *V*_*th*_ of TS-PCM in Fig. 3e is increased from 0.4 V to 0.6 V.

Synaptic plasticity is also emulated by nonvolatile characteristics of TS-PCM. Figure 3f describes the structure of synapse before and after the long-term potentiation (LTP), that explains the mechanism of synaptic plasticity in chemical synapse. Communication between adjacent neurons is regulated by the emission of neurotransmitters (blue dots) stored in synaptic vesicles of the pre-neuron axon terminal. The receptors in the post-neuron dendrite receive the released neurotransmitters, generating the excitatory postsynaptic potential (EPSP). Repetitive stimulations can increase the number of synaptic vesicles and receptors, increasing the connection strength and associated EPSP amplitude. Figure 3g shows an experimental demonstration of a symmetric Hebbian learning rule, one type of long-term synaptic plasticity. Same pulse conditions are applied for pre- and post-spike, using 0.6 V amplitude and 1 ms pulse width. The weight change is not accumulated since the device was reset between each measurement. The change in resistance of TS-PCM exhibits clear dependency on the time interval between pre-neuron and post-neuron spikes, the most basic form of a learning rule. The depression of synaptic weight is just as important as the potentiation process, due to the effect of causality on learning operation^58^. Based on the well-known phase change properties of GST film, the depression of synaptic weight is possible in TS-PCM cell (see Supplementary Fig. S8). In addition to long-term plasticity, emulation of short-term synaptic plasticity is demonstrated using volatile characteristics of TS-PCM. Figure 3h presents the emulation of paired-pulse facilitation, a well-known form of short-term synaptic plasticity. The output current of TS-PCM exhibits a temporary increase for two identical voltage pulses, due to the sufficiently small time interval. The finite retention time of Ag CF in the bottom TS layer leads to an increased filament radius for repetitive input, and this results in temporary increment of the output current value^59,60^. Short-term plasticity in artificial synapse provides enhanced learning capability such as working memory and decision operation, similar to biological systems.

### Intrinsic plasticity and synergistic concomitance

**Fig. 4:**
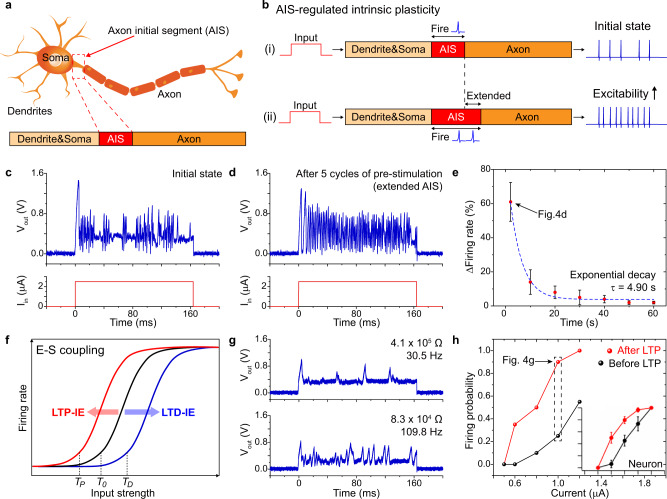
Intrinsic plasticity and synergistic concomitance in TS-PCM. **a** Illustration of neuron composed of dendrites, soma, and axon. A specific region of AIS is located between the soma and axon that integrates input signals into AP spikes. **b** Intrinsic plasticity by AIS structure. (i) AIS regulates the threshold of AP generation, modulating the firing frequency of the neuron. (ii) Elongation of AIS leads to extension of the excitable region, resulting in increment of the firing frequency. **c**, **d** Intrinsic plasticity of TS-PCM based on the *V*_*th*_ modulation in the bottom TS layer. Upon the same input current of 2.5 μA, the firing frequency increases from 287.5 Hz to 462.5 Hz by the application of pre-stimulations. Five cycles of pre-stimulations with a two second time interval prevents the complete diffusion of the Ag filament in the TS layer, leading to a decreased *V*_*th*_ and increased firing frequency. **e** Time interval dependency of intrinsic plasticity. The change of the firing rate exponentially decays with the increase of pre-stimulation time intervals, with a time coefficient of 4.90 s. Each data point indicates the mean value and the error bars indicate the standard deviation. **f** Schematic diagram of neuronal excitability curve with E-S coupling. The excitability curve is translated by the LTP and LTD of the synapse, presenting the synergistic interaction between intrinsic and synaptic plasticity. **g** Emulation of LTP-IE using TS-PCM based on the simultaneous implementation of synaptic and intrinsic plasticity. The firing frequency increases from 30.5 Hz to 109.8 Hz by the LTP of TS-PCM from 4.1 × 10^5^ Ω to 8.3 × 10^4^ Ω. **h** Intrinsic excitability curve of TS-PCM verifying the emulation of LTP-IE. Inset graph shows the excitability curve of a biological neuron. (Inset of Fig. 4h was reproduced with permission from [Campanac E. and Debanne D. Spike-timing-dependent plasticity: a learning rule for dendritic integration in rat CA1 pyramidal neurons.] ^66^. Copyright © 2008 The Physiological Society).

In addition to spiking behaviors, neuronal intrinsic plasticity should be emulated for the implementation of learning and memory functions. As shown in Fig. 4a, the biological neuron is composed of dendrites, soma, and axon, that govern signal reception, cell function, and signal transmission, respectively^61^. A specific region called the axon initial segment (AIS) is located between the soma and axon, where a large number of ion channels are concentrated^62^. As shown in Fig. 4b-(i), input signals are received by dendrites and transmitted to the axon through the AIS. Due to the concentrated ion channels, AIS is more excitable than other regions of the axon, regulating the threshold of AP generation in the neuron. The signal sensitivity of neuron in Fig. 4b-(i) is determined by the initial neuronal excitability, which is dependent on the length of AIS. As shown in 4b-(ii), the elongation of AIS expands the excitable region, increasing the intrinsic excitability of the neuron. The neuronal intrinsic plasticity, the ability of neuron to change its firing probability depending on the stimulation history, can be induced by the structural deformation of AIS region in neuron. The modulation of firing threshold by the structural change of AIS is known to be a key mechanism of intrinsic plasticity^63^.

The AIS-regulated excitability is similar to the *V*_*th*_ modulation of residual filament in the bottom TS layer, facilitating the emulation of intrinsic plasticity by TS-PCM. The reset process of the TS layer is based on the atomistic diffusion of Ag filament induced by the surface energy minimization. Residual filament of Ag clusters may remain after the spontaneous reset process, changing the threshold of filament formation. The *V*_*th*_ of TS-PCM is modulated depending on the size and location of filament residues, leading to a change of firing frequency in spiking emulation (see Supplementary Figs. S20 and S21). As shown in Fig. 4c, d, intrinsic plasticity is emulated by TS-PCM based on the *V*_*th*_ modulation of residual filament. After five cycles of pre-stimulation with a 2 s interval, the experimental firing frequency of TS-PCM increases from 287.5 Hz to 462.5 Hz for the same input current of 2.5 μA. Note that experimental spike frequency has been calculated from the number of voltage spikes per input pulse width. Pre-stimulation with short time interval prevents the complete diffusion of Ag filament, leading to lowered *V*_*th*_ and increased excitability. As shown in Fig. 4e, the change of excitability is dependent on the pre-stimulation intervals. An exponential decaying function is fitted to the intrinsic plasticity curve of TS-PCM:

5 $$y=A\cdot {{{{{\rm{exp }}}}}}\left(-\frac{x}{\tau }\right)+{y}_{0}$$

The excitability exponentially increases as the time interval decreases below a *τ* of 4.90 s, due to the reduction of filament diffusion time. Figure 4c–e indicate that TS-PCM can emulate the neuronal intrinsic plasticity, specifically the structural AIS plasticity-induced excitability change in biological neuron. Neuron intrinsic plasticity can be classified into three categories: EPSP amplification, spike threshold modulation, and resting potential change^1^. Spike threshold of neuron can be modulated by the AIS structural plasticity, which is analogous to the *V*_*th*_ modulation by the Ag residual filament adaptability. The elongation of AIS in the neuron can be induced by specific stimulation patterns, which confirms high similarity between TS-PCM and a biological system^64^.

During the biological learning process, synaptic and intrinsic plasticity occur interdependently with synergistic interactions. Figure 4f describes the effect of long-term synaptic plasticity on neuronal excitability, called EPSP-spike coupling (E-S coupling)^4,65,66^. E-S coupling is one of the main mechanisms of intrinsic plasticity, which is induced by the synaptic weight change instead of AIS structural plasticity. The initial excitability curve (black) follows a sigmoid function with a threshold *T*_*0*_, which shows horizontal translations depending on the synaptic modifications. Increased synaptic strength reduces the threshold to *T*_*P*_, resulting in long-term potentiation of intrinsic plasticity (LTP-IE). As shown in Fig. 4g, LTP-IE is implemented in TS-PCM based on the concomitant emulation of synaptic and intrinsic plasticity. The resistance of TS-PCM decreases from 4.1 × 10^5^ Ω to 8.3 × 10^4^ Ω by resistive switching of the top PCM layer, leading to increment of the experimental spiking frequency from 30.5 Hz to 109.8 Hz. There are delay time between the voltage spikes for the high resistance state, which is induced by the increased relaxation time of the Ag filament. The value of *V*_*min*_ increases with the increment of device resistance, which hinders the diffusion of the conductive filament. As a result, the experimental spiking frequency deviates from the voltage spiking equation. Note that the spiking behavior of TS-PCM is inherently stochastic, showing random noise patterns and chaotic deviations from the voltage spiking equation. The inherent stochasticity of TS-PCM can be originated from the thermal fluctuation that induces the distribution of diffusion time of Ag ions^67–69^. Figure 4h demonstrates the excitability curve of TS-PCM, presenting the horizontal translation after the LTP process. The excitability curve of the hippocampal neuron (CA1 region) is shown in the inset of Fig. 4h^66^, verifying that TS-PCM successfully mimicked E-S coupling behavior in biological neuron. The emulation of E-S coupling indicates that TS-PCM implements the synergistic interactions between synaptic and intrinsic plasticity, promoting the realization of complicated learning functions.

### Concomitant neuroplasticity and feedback learning in TS-PCM

**Fig. 5:**
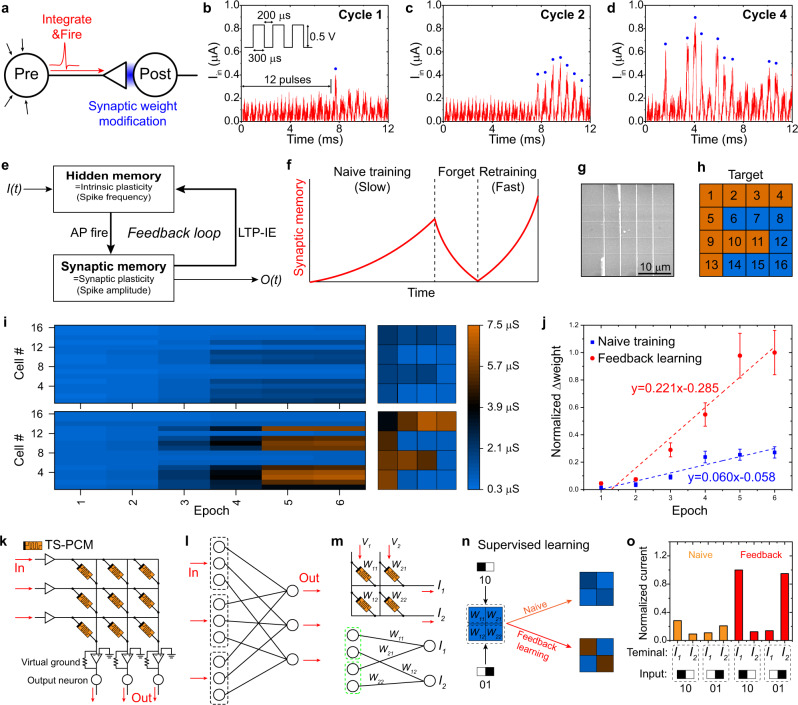
Concomitant plasticity and positive feedback learning in TS-PCM. **a** Illustration of neuron-synapse pair structure. Input signals are integrated to fire an AP spike modulating the synaptic strength. **b**–**d** Emulation of basic learning process using TS-PCM with the LIF model. Each cycle is consisted of 20 voltage pulses. For a repetitive input pulse trains, the number of current spikes increases along with the maximum current value, indicating the concomitant implementation of synaptic and intrinsic plasticity. Note that small peaks around 0.2 μA are the displacement current caused by the voltage gradient, which do not represent actual movement of charges across the cell. **e** Schematic diagram of positive feedback learning loop based on the concomitant neuroplasticity and LTP-IE. Intrinsic plasticity and synaptic plasticity construct hidden memory and synaptic (regular) memory states, respectively. **f** Illustration of naive training and retraining, presenting a clear difference in the rate of acquisition. Hidden memory is developed during naive training and remains after the forget process, promoting reinforcement of the retraining process. **g** SEM image of crossbar structured 4 × 4 TS-PCM array. **h** 4 × 4 pixel image of “F” used in pattern memorization task in **i** and **j**. **i** Resistance-based color map of naive training (upper panel) and feedback learning (lower panel) for total 6 training epochs. The right panels show the resistance states of the TS-PCM array after the training process. **j** Normalized synaptic weight updates for two different training schemes. Each data point indicates the mean value and the error bars indicate the standard deviation. **k** Schematic of TS-PCM array consists of TS-PCM, post-neuron device, and peripheral circuitry. **l** Network structure of TS-PCM array indicating that each word line pre-neurons are subdivided according to the number of bit lines. **m** Schematic and network structure of 2 × 2 TS-PCM array. **n** Conductance level before and after the supervised training process. Two types of 2 × 1 binary patterns were trained in the array with naive and feedback training schemes. **o** pattern classification result in TS-PCM array. Each pattern can be recognized by the output word line current level.

In the learning and memory mechanism of biological neural network, synaptic strength is modified by neuronal AP spikes. As shown in Fig. 5a, input stimuli are integrated in the pre-neuron to fire AP spikes, subsequently modifying the synaptic weight depending on the pattern of generated AP spikes. TS-PCM emulates the biological learning process based on the concomitant neuroplasticity. Figure 5b–d demonstrate the emulation of basic learning process using the LIF behavior of TS-PCM (see Supplementary Fig. S22). As shown in the inset of Fig. 5b, total 20 voltage pulses with 0.5 V amplitude is equally applied from cycle 1 to cycle 4, which is lower than *V*_*th*_ of bottom TS layer. Despite of the small input amplitude, a current spike (blue dot) of 83.3 Hz and 0.40 μA is generated after 13 voltage pulses in cycle 1 of Fig. 5b, due to the emulation of LIF neuron model. In cycle 2 of Fig. 5c, spike frequency and amplitude simultaneously increase to 583.3 Hz and 0.51 μA upon same input pulse train. A consecutive stimulation promotes the modulation of firing frequency and synaptic weight, due to the emulation of intrinsic and synaptic plasticity, respectively. The spike frequency and amplitude further increase in cycle 4 of Fig. 5d, showing highest value of 750.0 Hz and 0.85 μA. The simultaneous increment of firing frequency and spike amplitude indicates the concomitant intrinsic and synaptic plasticity, mimicking learning mechanism of neuron-synapse pair structure shown in Fig. 5a. The concomitant neuroplasticity has also been confirmed in single voltage pulse input, confirming the coexistence of neuronal spiking, intrinsic plasticity, and synaptic plasticity (see Supplementary Fig. S23).

A positive feedback learning loop is established in TS-PCM based on the concomitant neuroplasticity and LTP-IE. As illustrated in Fig. 5e, intrinsic and synaptic plasticity participate in the learning process, forming hidden and synaptic memory states, respectively. The input signal *I(t)* is integrated in the neuron to fire AP spikes modulating the synaptic memory state. The enhanced synaptic strength increases the intrinsic plasticity by LTP-IE, leading to recording of the hidden memory state. In this feedback loop, hidden memory regulates the state of synaptic memory by AP fire, and synaptic memory also change the state of hidden memory by LTP-IE. The AP spiking frequency is subsequently increased by hidden memory, reinforcing the synaptic modification process. The concomitant neuroplasticity and LTP-IE promote positive feedback between the neuron and synapse, leading to an increased rate of acquisition. For example, retraining in the biological system is faster than naive training due to the increased excitability, emphasizing the role of intrinsic plasticity in the learning process^70^. A schematic illustration in Fig. 5f describes naive training, forgetting, and retraining process, that shows a clear difference in the learning rate^4,26^. The synaptic memory state is updated by consecutive stimulation in naive training, presenting relatively slow rate of acquisition compared to retraining process. The intrinsic excitability of the neuron is increased during naive training, forming the hidden memory state that remains after the forgetting process. The retraining process is subsequently boosted by the hidden memory as shown in the feedback loop of Fig. 5e, that results in the faster rate of acquisition.

The positive feedback learning is demonstrated using a TS-PCM array based on the emulation of concomitant neuroplasticity. As shown in the SEM image of Fig. 5g, a crossbar structured 4 × 4 TS-PCM array is fabricated to conduct a pattern memorization task. A 4 × 4 pixel image of “F” in Fig. 5h is trained in TS-PCM array using two different training schemes of naive training and feedback learning. To examine the effect of positive feedback on weight update process in each cell, a single pattern without noise is utilized for the learning operations. A 0.5 V training pulse is applied in the orange-colored pixels for synaptic memory updates and pattern memorization, while 0.1 V noise pulse is applied in the blue-colored pixels. Figure 5i illustrates the conductance-based color map of 16 TS-PCM cells with increasing training epochs for naive training (upper panel) and feedback learning (lower panel). A 0.5 V training pulse is iteratively applied for six epochs in both learning schemes, resulting in the memorization of letter “F” as shown in the right panels of Fig. 5i. For the feedback learning, five cycles of 0.3 V pre-training pulse is applied prior to the training epochs to develop hidden memory states. Note that synaptic memory states remain unchanged after the pre-training epochs, as shown in the conductance level of feedback learning in epoch 1. In other words, the pre-training voltage 0.3 V is large enough to activate the filament formation in TS layer, but is small to initiate the phase transition in PCM layer. Through the positive feedback loop, the hidden memory reinforces the learning process, leading to the accelerated acquisition in feedback learning. As illustrated in the lower panel of Fig. 5i, the average conductance level of the TS-PCM cells in feedback learning quickly diverges from 0.64 μS to 5.60 μS, presenting faster learning rate compared to naive training (see also Supplementary Fig. S24). Figure 5j summarizes the synaptic weight updates of two different training schemes with linear fitting functions. The acquisition rate of each training process is calculated as 0.06/epoch and 0.221/epoch for naive training and feedback learning, respectively. In feedback learning, pre-training stimulation induces the increment of excitability and firing frequency, leading to the three-fold faster rate of synaptic weight modification. It is noteworthy that the acquisition rate of naive training can increase after a few more training epochs, since the intrinsic excitability of TS-PCM increases during the training. Because of the one-to-one correspondence between pre-neuron and synapse, the network structure of TS-PCM array appears to be independent connections of each cells. However, TS-PCM array is more like subdividing a pre-neuron in a word line into multiple pre-neuron axon terminal in associate bit line as illustrated in Fig. 5k. The multiple bit line pre-neuron axon terminals, the TS layers in TS-PCM, are still tied together in each word lines, enabling learning operations like other memristive neural networks as shown in Fig. 5l. Note that the output post-neuron devices are connected to the TS-PCM array followed by the virtual ground circuits^71^. As presented in Fig. 5m, the accelerated training can be applied to learning operation in TS-PCM based ANN since the positive feedback effect have been confirmed in weight update of each device. Simple binary patterns of “10” and “01” are input to 2 × 2 TS-PCM array with two different training schemes of naive and feedback learning, as shown in Fig. 5n^72^. The binary patterns can be identified through the bit line currents *I*_*1*_ and *I*_*2*_, following the simple equation of ${I}_{j}=\sum {V}_{i}{W}_{{ij}}$. Figure 5o shows the current response of TS-PCM array with two different input binary patterns. The orange-colored bar graphs of naive training confirm that each bit line identifies different patterns, “10” for *I*_*1*_ and “01” for *I*_*2*_, respectively. The red-colored graphs of feedback training shows amplified current output compared to that of naive training, presenting the accelerated acquisition in supervised learning operation.

## Discussion

We proposed a memristive synaptic device composed of volatile TS and nonvolatile PCM layers in a single element. On the basis of the functional similarity, tonic spiking and bursting were successfully demonstrated by the RC circuit of TS-PCM. Synaptic plasticity was confirmed by the mimicking of a symmetric Hebbian learning rule and paired-pulse facilitation, verifying the successful emulation of a synapse by TS-PCM. In addition, neuronal intrinsic plasticity was achieved based on the analogy between the AIS of neuron and the residual filament of bottom TS layer, presenting the increment of spiking frequency by pre-stimulation. The LTP-IE of TS-PCM was enabled by the synergistic concomitance of synaptic and intrinsic plasticity, showing a similar excitability curve to that of the biological neuron. A positive feedback learning loop is established in TS-PCM so that hidden memory modulates the state of synaptic memory and vice versa. The feedback learning process is demonstrated in a crossbar structured 4 × 4 TS-PCM array, which exhibits a three-fold higher acquisition rate compared to naive training similar to the biological retraining process. TS-PCM provides a concomitant solution for the hardware realization of an artificial synapse with synergistic interactions, presenting high similarity with the complicated learning mechanism of biological system. Further studies on homeostatic plasticity and functional stability can promote a more comprehensive emulation of the neural network for brain-inspired neuromorphic computing.

## Methods

### Fabrication of TS-PCM device

A 8 inch silicon wafer was prepared was used for the fabrication of TS-PCM. A 100 nm × 20 μm size line pattern was generated via EBL process using JEOL JBX-9300FS and e-beam resist PMMA. A 5 nm thick Ti adhesion layer and a 30 nm thick Au bottom electrode were deposited by e-beam evaporator, followed by a lift-off process. A 100 μm × 100 μm size bottom contact pad was patterned by conventional photolithography process. A 20 nm thick Ti adhesion layer and a 40 nm thick Au layer were subsequently deposited by e-beam evaporator and were lift-off in ultrasonic bath with acetone. The switching medium of bottom TS layer was deposited by sputtering of a 1 nm thick Ag layer and a 15 nm thick Ag:SiO_2_ layer. For the deposition of Ag:SiO_2_, Ag and SiO_2_ targets were co-sputtered in Ar atomosphere. A 100 nm × 20 μm size top line pattern was generated via EBL process using VEGA3 ELPHY Quantum Lithography System and e-beam resist PMMA. A 10 nm thick phase changing GST layer was deposited by RF sputtering of GST target in Ar atmosphere, followed by the sputtering of a 40 nm thick TiW layer. A nanopattern PMMA film was lift-off in ultrasonic bath with acetone. A top contact pad was patterned via conventional lithography process, followed by the deposition of a TiW film and lift-off process.

### Device characterization

All electrical measurements were conducted using a Keithley 4200-SCS semiconductor parameter analyzer. Keithley 4225-PMU with a remote amplifier/switch (Keithley 4225-RPM) was also utilized for the voltage pulse measurements. In the spiking emulations, voltage spikes were measured by Tektronix DPO 3054 digital phosphor oscilloscope with P6139B voltage probe (10 MΩ input resistance). A conventional ceramic capacitor of 4.72 nF and 5.61 nF capacitance were connected in parallel for the TS-PCM RC circuit.

## Supplementary information

Supplementary Information

## Data Availability

The data that support the findings of this study are present in the article and Supplementary Information. Additional data related to this study is available from the corresponding author upon request.

## References

[1] Debanne D, Inglebert Y, Russier M (2019). Plasticity of intrinsic neuronal excitability. Curr. Opin. Neurobiol..

[2] Frick A, Johnston D (2005). Plasticity of dendritic excitability. J. Neurobiol..

[3] Sehgal M, Song C, Ehlers VL, Moyer JR (2013). Learning to learn—intrinsic plasticity as a metaplasticity mechanism for memory formation. Neurobiol. Learn. Mem..

[4] Mozzachiodi R, Byrne JH (2010). More than synaptic plasticity: role of nonsynaptic plasticity in learning and memory. Trends Neurosci..

[5] Zhang W, Linden DJ (2003). The other side of the engram: experience-driven changes in neuronal intrinsic excitability. Nat. Rev. Neurosci..

[6] Zidan MA, Strachan JP, Lu WD (2018). The future of electronics based on memristive systems. Nat. Electron..

[7] Berggren K (2020). Roadmap on emerging hardware and technology for machine learning. Nanotechnology.

[8] Ghoneim MT, Zidan MA, Salama KN, Hussain MM (2014). Towards neuromorphic electronics: Memristors on foldable silicon fabric. Microelectron. J..

[9] Sung SH (2021). Memory-centric neuromorphic computing for unstructured data processing. Nano Res..

[10] Strukov DB, Snider GS, Stewart DR, Williams RS (2008). The missing memristor found. Nature.

[11] Ghoneim MT, Hussain MM (2015). Review on physically flexible nonvolatile memory for internet of everything electronics. Electronics.

[12] Li Y (2021). Anomalous resistive switching in memristors based on two-dimensional palladium diselenide using heterophase grain boundaries. Nat. Electron..

[13] Shi Y (2018). Electronic synapses made of layered two-dimensional materials. Nat. Electron..

[14] Ohno T (2011). Short-term plasticity and long-term potentiation mimicked in single inorganic synapses. Nat. Mater..

[15] Prezioso M (2018). Spike-timing-dependent plasticity learning of coincidence detection with passively integrated memristive circuits. Nat. Commun..

[16] Wang Z (2018). Fully memristive neural networks for pattern classification with unsupervised learning. Nat. Electron..

[17] Yi W (2018). Biological plausibility and stochasticity in scalable VO 2 active memristor neurons. Nat. Commun..

[18] Wang Z (2018). Capacitive neural network with neuro-transistors. Nat. Commun..

[19] Duan Q (2020). Spiking neurons with spatiotemporal dynamics and gain modulation for monolithically integrated memristive neural networks. Nat. Commun..

[20] Woo J, Wang P, Yu S (2019). Integrated crossbar array with resistive synapses and oscillation neurons. IEEE Electron Device Lett..

[21] Pantazi A, Woźniak S, Tuma T, Eleftheriou E (2016). All-memristive neuromorphic computing with level-tuned neurons. Nanotechnology.

[22] Wang Z (2019). Reinforcement learning with analogue memristor arrays. Nat. Electron..

[23] Lin P (2020). Three-dimensional memristor circuits as complex neural networks. Nat. Electron..

[24] Kim H, Mahmoodi M, Nili H, Strukov DB (2021). 4K-memristor analog-grade passive crossbar circuit. Nat. Commun..

[25] Yoon JH (2018). An artificial nociceptor based on a diffusive memristor. Nat. Commun..

[26] Baek E (2020). Intrinsic plasticity of silicon nanowire neurotransistors for dynamic memory and learning functions. Nat. Electron..

[27] Guo T (2018). Overwhelming coexistence of negative differential resistance effect and RRAM. Phys. Chem. Chem. Phys..

[28] Wang Y (2021). Artificial Neurons Based on Ag/V_2_C/W Threshold Switching Memristors. Nanomaterials-Basel.

[29] Wang Y (2021). Emulation of multiple-functional synapses using V_2_C memristors with coexistence of resistive and threshold switching. Mat. Sci. Semicon. Proc..

[30] Wang ZR (2017). Memristors with diffusive dynamics as synaptic emulators for neuromorphic computing. Nat. Mater..

[31] You BK, Byun M, Kim S, Lee KJ (2015). Self-Structured Conductive Filament Nanoheater for Chalcogenide Phase Transition. ACS Nano.

[32] Suh, D.-S. et al. Critical Quenching Speed Determining Phase of Ge_2_Sb_2_Te_5_ in Phase-Change Memory. 2006 International Electron Devices Meeting; 2006. 1–4. (IEEE, 2006).

[33] Wang W (2019). Surface diffusion-limited lifetime of silver and copper nanofilaments in resistive switching devices. Nat. Commun..

[34] Stoneham A (1983). Systematics of metal-insulator interfacial energies: A new rule for wetting and strong catalyst-support interactions. Appl. Surf. Sci..

[35] Stoliar P (2017). A leaky‐integrate‐and‐fire neuron analog realized with a Mott insulator. Adv. Funct. Mater..

[36] Lee D (2019). Various threshold switching devices for integrate and fire neuron applications. Adv. Electron. Mater..

[37] Zhu M (2014). One order of magnitude faster phase change at reduced power in Ti-Sb-Te. Nat. Commun..

[38] Khan AI (2021). Ultralow-switching current density multilevel phase-change memory on a flexible substrate. Science.

[39] Ascoli A, Slesazeck S, Mahne H, Tetzlaff R, Mikolajick T (2015). Nonlinear Dynamics of a Locally-Active Memristor. IEEE Trans. Circuits Syst. I: Regul. Pap..

[40] Bohaichuk SM (2019). Fast Spiking of a Mott VO2-Carbon Nanotube Composite Device. Nano Lett..

[41] Messaris, I. et al. A Simplified Model for a NbO_2_ Mott Memristor Physical Realization. in 2020 IEEE International Symposium on Circuits and Systems (ISCAS), 1-5 (IEEE, 2020).

[42] Zhuo, Y. et al. A Dynamical Compact Model of Diffusive and Drift Memristors for Neuromorphic Computing. Adv. Electron. Mater. 2100696 (2021).

[43] Ventrice D (2007). A phase change memory compact model for multilevel applications. IEEE Electron Device Lett..

[44] Zidan MA, Fahmy HAH, Hussain MM, Salama KN (2013). Memristor-based memory: The sneak paths problem and solutions. Microelectron. J..

[45] Kim DH (2019). Flexible Crossbar-Structured Phase Change Memory Array via Mo-Based Interfacial Physical Lift-Off. Adv. Funct. Mater..

[46] Huang C-H, Chou T-S, Huang J-S, Lin S-M, Chueh Y-L (2017). Self-Selecting Resistive Switching Scheme Using TiO2 Nanorod Arrays. Sci. Rep..

[47] Gül F (2019). Addressing the sneak-path problem in crossbar RRAM devices using memristor-based one Schottky diode-one resistor array. Results Phys..

[48] Kumar S, Williams RS, Wang Z (2020). Third-order nanocircuit elements for neuromorphic engineering. Nature.

[49] Breitenstein C (2006). Tonic dopaminergic stimulation impairs associative learning in healthy subjects. Neuropsychopharmacol.

[50] Lee S, Shin HS (2016). The role of mediodorsal thalamic nucleus in fear extinction. J. Anal. Sci. Technol..

[51] Weyand TG, Boudreaux M, Guido W (2001). Burst and tonic response modes in thalamic neurons during sleep and wakefulness. J. Neurophysiol..

[52] Metzen MG, Krahe R, Chacron MJ (2016). Burst Firing in the Electrosensory System of Gymnotiform Weakly Electric Fish: Mechanisms and Functional Roles. Front. Comput. Neurosci..

[53] Doron G (2020). Perirhinal input to neocortical layer 1 controls learning. Science.

[54] Kumar, A., Kansal, S. & Hanmandlu M. Classification of different neuron behavior by designing spiking neuron model. In 2013 IEEE International Conference ON Emerging Trends in Computing, Communication and Nanotechnology (ICECCN); 2013: p. 25–30 (IEEE, 2013).

[55] Han Y (2020). Excitatory VTA to DH projections provide a valence signal to memory circuits. Nat. Commun..

[56] Golowasch J (2009). Membrane Capacitance Measurements Revisited: Dependence of Capacitance Value on Measurement Method in Nonisopotential Neurons. J. Neurophysiol..

[57] Gentet LJ, Stuart GJ, Clements JD (2000). Direct measurement of specific membrane capacitance in neurons. Biophys. J..

[58] Zappacosta S, Mannella F, Mirolli M, Baldassarre G (2018). General differential Hebbian learning: Capturing temporal relations between events in neural networks and the brain. PLOS Comput. Biol..

[59] Song M-J, Kwon K-H, Park J-G (2017). Electro-forming and electro-breaking of nanoscale Ag filaments for conductivebridging random-access memory cell using Ag-doped polymer-electrolyte between Pt electrodes. Sci. Rep..

[60] Yang Y (2012). Observation of conducting filament growth in nanoscale resistive memories. Nat. Commun..

[61] Won SM, Song E, Reeder JT, Rogers JA (2020). Emerging Modalities and Implantable Technologies for Neuromodulation. Cell.

[62] Huang CYM, Rasband MN (2018). Axon initial segments: structure, function, and disease. Ann. Ny. Acad. Sci..

[63] Grundemann J, Hausser M (2010). NEUROSCIENCE A plastic axonal hotspot. Nature.

[64] Booker SA (2020). Input-Output Relationship of CA1 Pyramidal Neurons Reveals Intact Homeostatic Mechanisms in a Mouse Model of Fragile X Syndrome. Cell Rep..

[65] Daoudal G, Hanada Y, Debanne D (2002). Bidirectional plasticity of excitatory postsynaptic potential (EPSP)-spike coupling in CA1 hippocampal pyramidal neurons. Proc. Natl Acad. Sci..

[66] Campanac E, Debanne D (2008). Spike timing-dependent plasticity: a learning rule for dendritic integration in rat CA1 pyramidal neurons. J. Physiol..

[67] Kumar S, Strachan JP, Williams RST (2017). Chaotic dynamics in nanoscale NbO2 Mott memristors for analogue computing. Nature.

[68] Carboni R, Ielmini D (2019). Stochastic Memory Devices for Security and Computing. Adv. Electron. Mater..

[69] Ushakov Y, Balanov A, Savel’ev S (2021). Role of noise in spiking dynamics of diffusive memristor driven by heating-cooling cycles. Chaos Soliton Fract..

[70] Medina JF, Garcia KS, Mauk MD (2001). A mechanism for savings in the cerebellum. J. Neurosci..

[71] Woods, W. & Teuscher, C. Approximate Vector Matrix Multiplication Implementations for Neuromorphic Applications using Memristive Crossbars. in 2017 IEEE/ACM International Symposium on Nanoscale Architectures (NANOARCH), 103-108 (IEEE, 2017).

[72] Feldmann J, Youngblood N, Wright CD, Bhaskaran H, Pernice WHP (2019). All-optical spiking neurosynaptic networks with self-learning capabilities. Nature.

