# Supplementary material for: Simultaneous emulation of synaptic and intrinsic plasticity using a memristive synapse
Source: Nat Commun. 2022 May 19;13:2811. doi: 10.1038/s41467-022-30432-2 (PMC9120471; doi:10.1038/s41467-022-30432-2)
Supplement: Supplementary file 1 — Supplementary Information [file 41467_2022_30432_MOESM1_ESM.pdf]

# Supplementary Information

**Simultaneous emulation of synaptic and intrinsic plasticity using a memristive synapse**

*Sang Hyun Sung, Tae Jin Kim, Hyera Shin, Tae Hong Im†, and Keon Jae Lee**

S. H. Sung, T. J. Kim, H. Shin, T. H. Im, and Prof. K. J. Lee
Department of Materials Science and Engineering, Korea Advanced Institute of Science and Technology (KAIST)
291 Daehak-ro, Yuseong-gu, Daejeon
34141, Republic of Korea
E-mail: keonlee@kaist.ac.kr

†Current Address: Memory Division, Samsung Electronics Co. Ltd.
Pyeongtaek-si, Gyeonggi-do, South Korea

**Keywords:** neuromorphic computing, intrinsic plasticity, concomitant plasticity, threshold switch, phase change memory

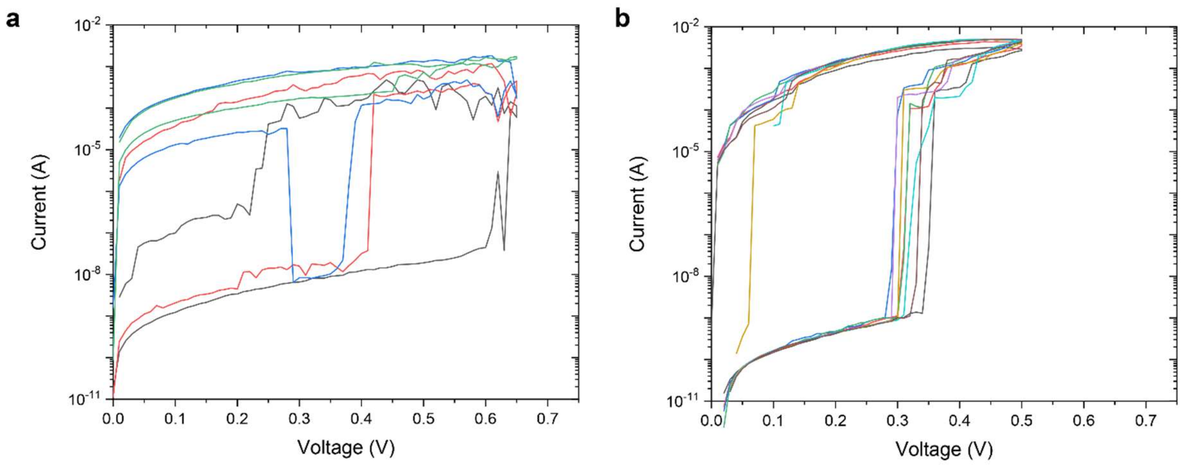


**Fig. S1 | Effect of a 1 nm thick Ag layer in TS device**. **a**, I-V curve of TS device without a Ag seed layer, showing unstable resistive switching properties. **b**, I-V curve of TS device with a 1 nm thick Ag seed layer which stabilizes the formation of Ag filament.

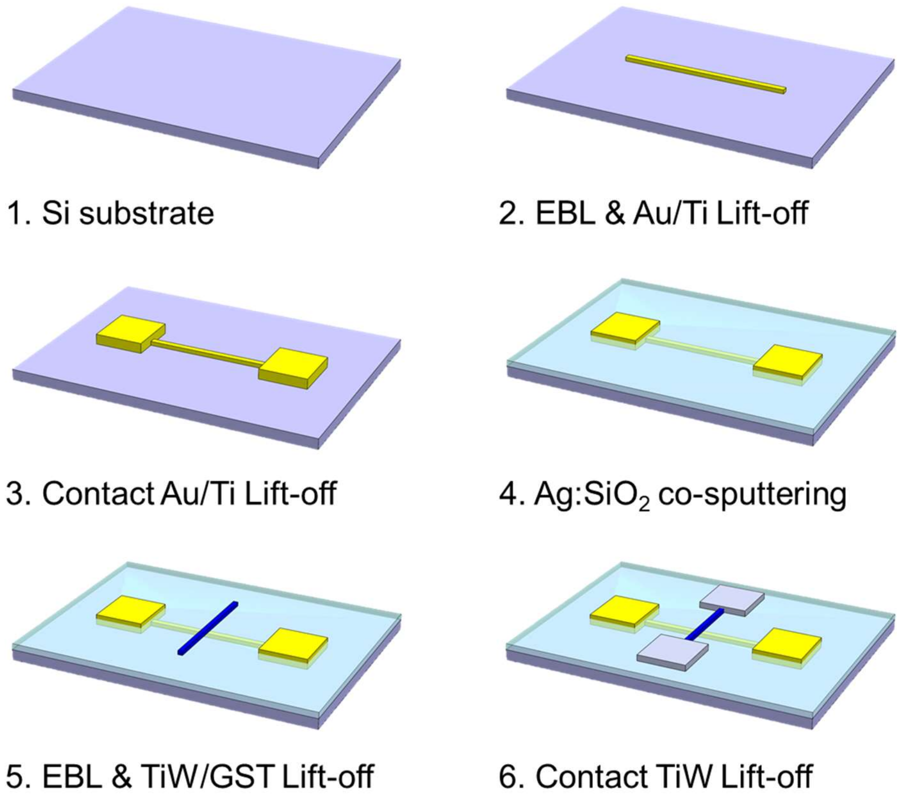


**Fig. S2 | Fabrication process of TS-PCM cell**. A Si substrate is utilized for the fabrication of TS-PCM. An e-beam lithography (EBL) process is carried out for the patterning of bottom electrode (Au/Ti). The SEM image of nanopatterned bottom electrode is shown in supplementary Fig. S3. Bottom contact pad is patterned by conventional photolithography process, followed by the deposition and lift-off process. A threshold switching layer Ag:$SiO_2$ film is deposited by the co-sputtering of Ag and Si targets in Ar atmosphere at 5 mTorr. Top electrode is also patterned by EBL to minimize the contact area and stabilize the formation/dissolution volatile Ag filament. Phase changing layer GST and electrode layer TiW are deposited and lift-off for top PCM layer. Lastly, top contact pads are patterned by conventional lithography process, followed by the sputtering of TiW and lift-off process.

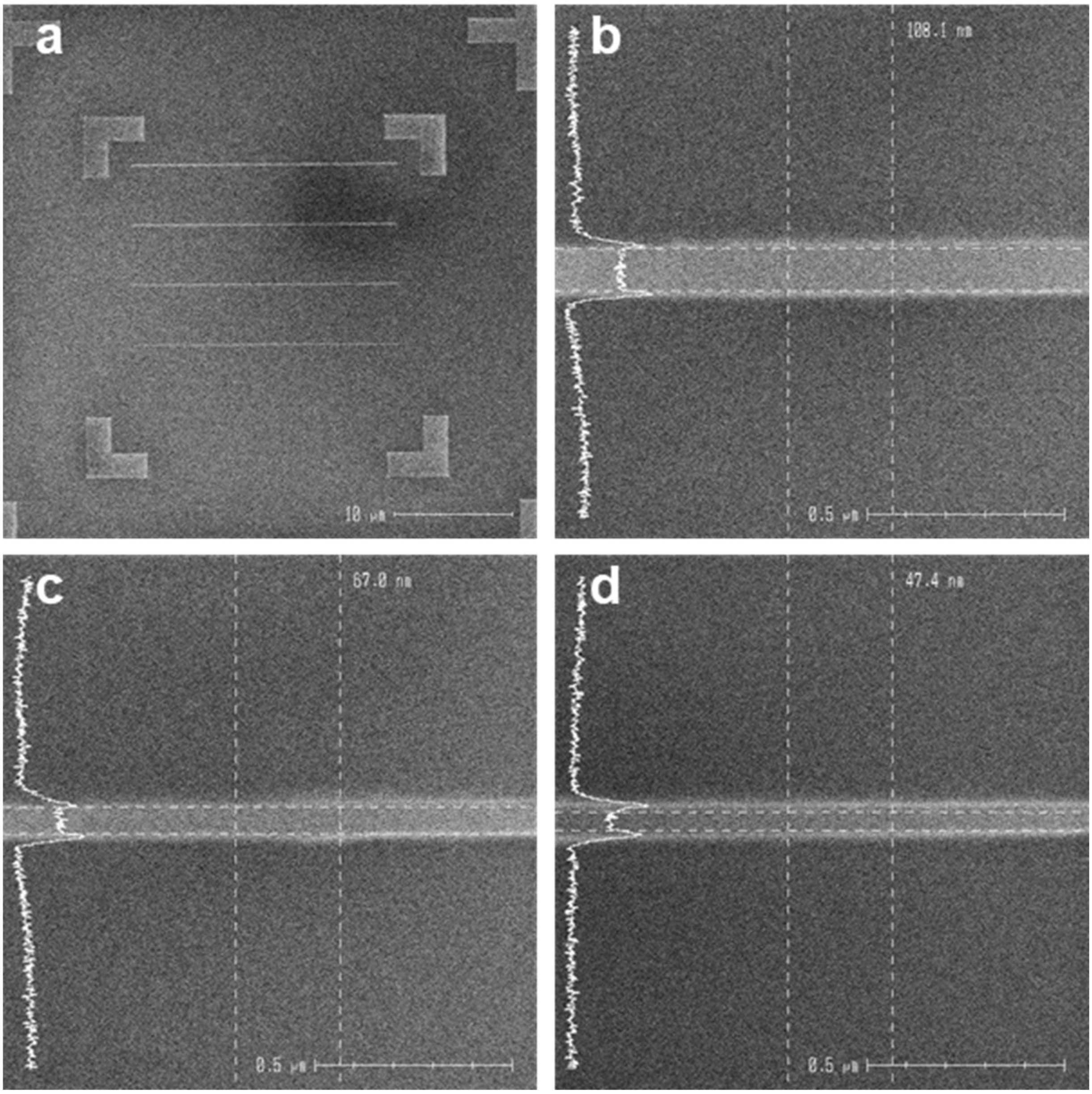


**Fig. S3 | Nanopatterning by EBL process.** **a**, SEM image of nanopatterned lines for inspection. **b**, A 108.1 nm width nanopatterned Au/Ti electrode. **c**, A 67.0 nm width nanopatterned electrode. **d**, A 47.4 nm width nanopatterned electrode. Note that a 108.1 nm width line was utilized for the fabrication of TS-PCM.

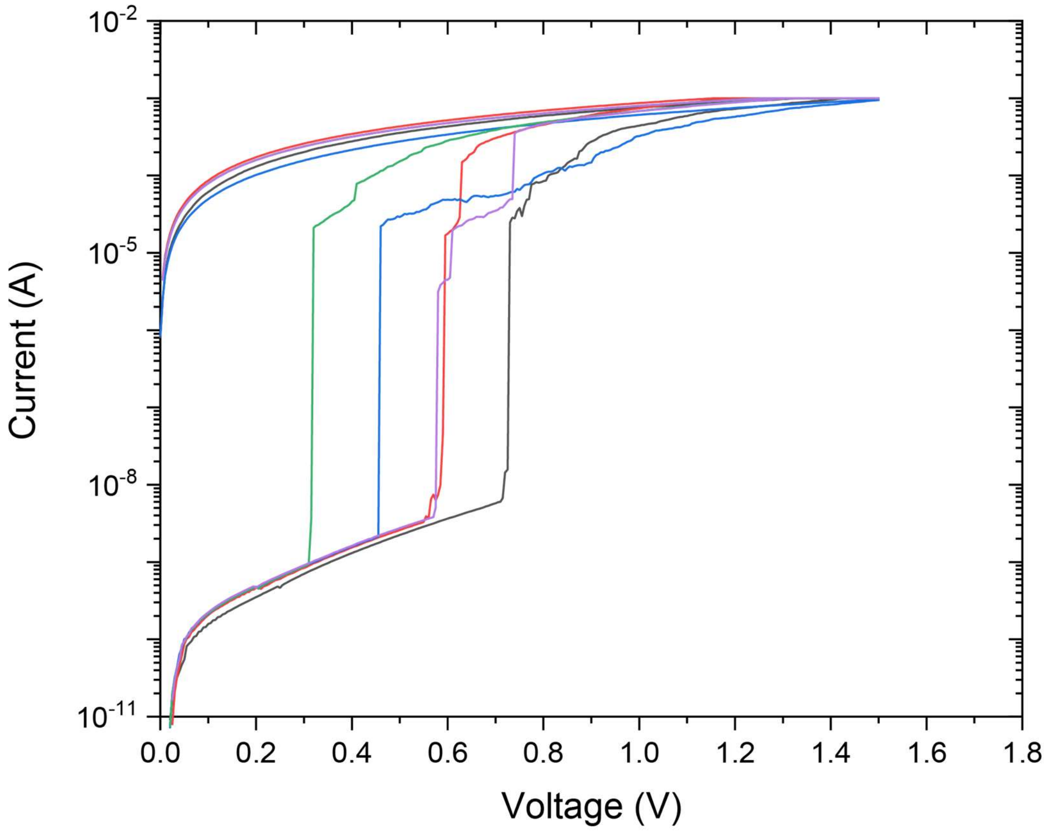


**Fig. S4 | Micro-size TS-PCM device fabricated by conventional lithography process.** The I-V curve of micro-size TS-PCM shows threshold switching behavior without nonvolatile resistance changes. Due to the randomness and stochasticity of Ag filament formation, micro-size TS-PCM presents only volatile resistive switching. Note that each voltage sweep is measured after the application of reset pulse, which fails to change the resistance of GST film.

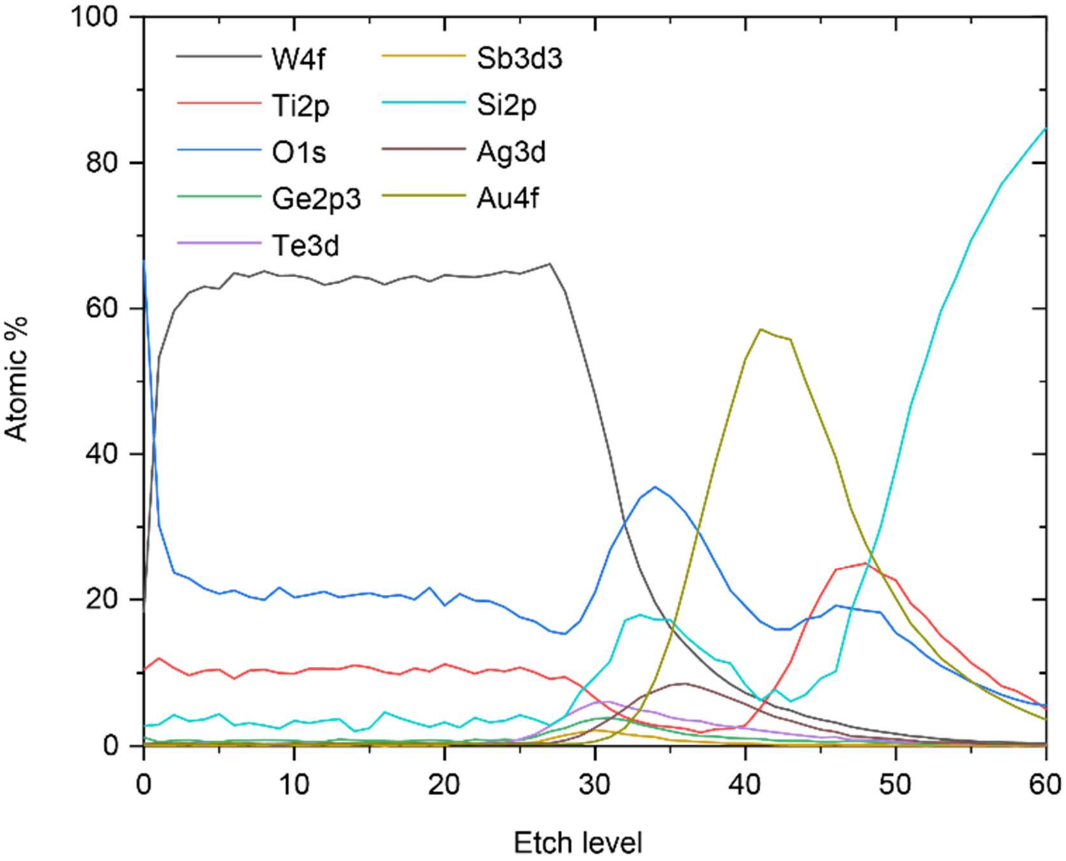


**Fig. S5 | X-ray photoelectron spectroscopy (XPS) depth profile of TS-PCM**. The layered structure of TS-PCM is clearly demonstrated in XPS depth profile. The profile of Ag shows accumulation at $SiO_2$/Au interface due to the 1 nm thick Ag seed layer of $V_{th}$ stabilization. The profiles of Ge, Sb, and Te are not significant because of the small thickness of GST film (<10 nm).

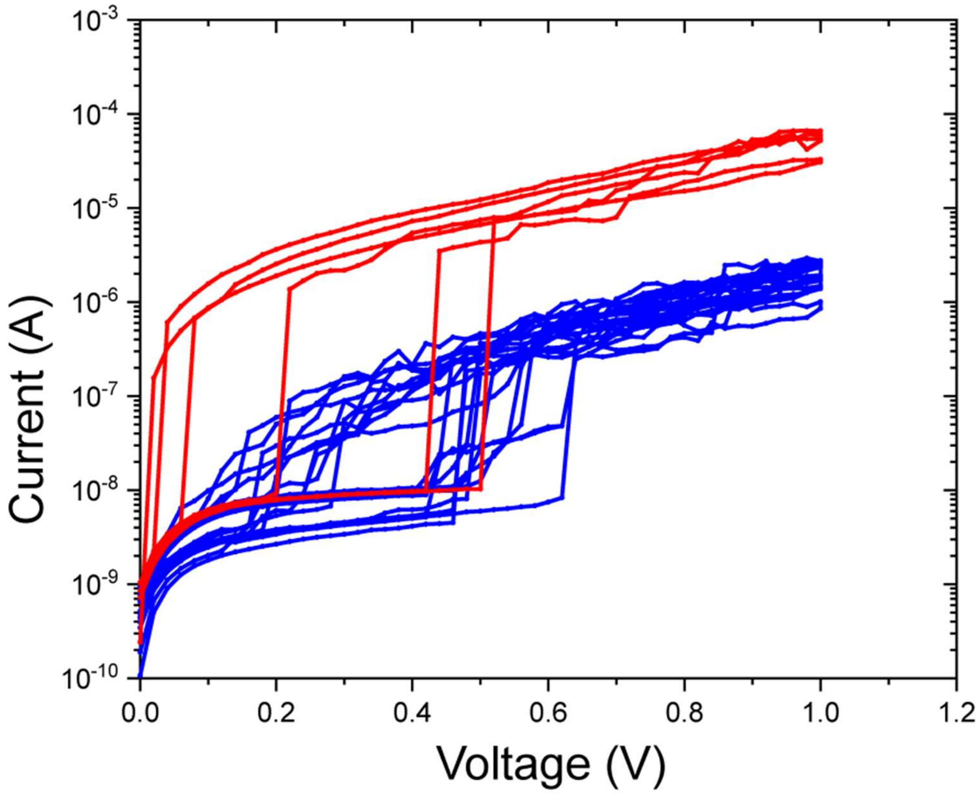


**Fig. S6 | I-V curve of TS-PCM cell**. The I-V graph clearly demonstrates the low resistance state (red) and high resistance state (blue) of TS-PCM modulated by the phase of GST layer. Note that voltage sweep in high resistance state can be repetatively measured, verifying that low resistance state of TS-PCM does not originate from the morphology of voltaile Ag filament in bottom TS layer.

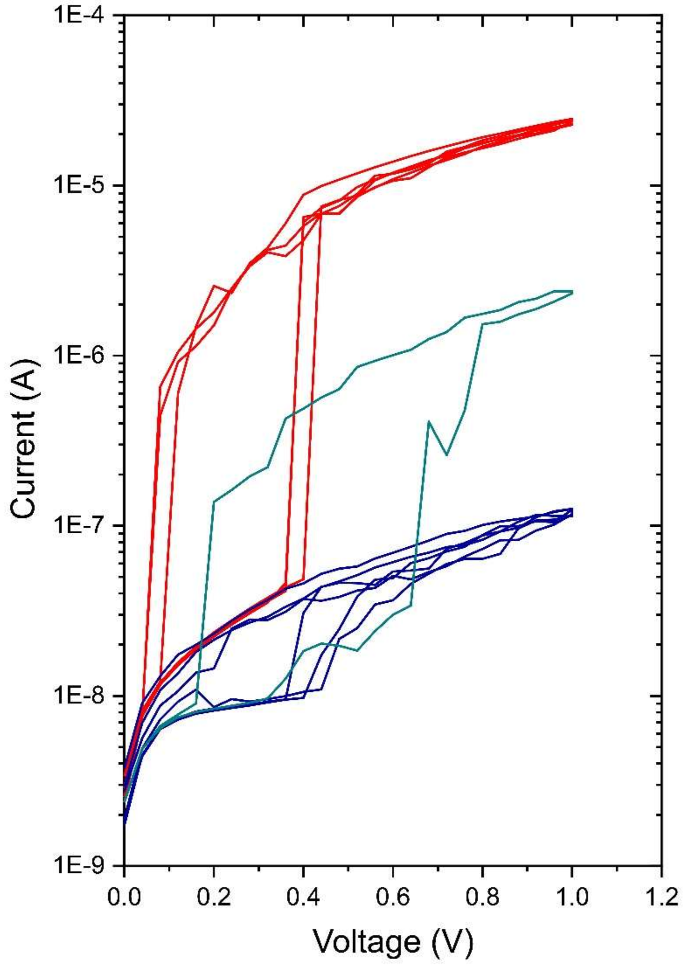


**Fig. S7 | Intermediate resistance state of TS-PCM**. The I-V curve shows that an intermediate resistance state can be acheived in TS-PCM cell, as demonstrated in cyan-colored curve. An intermediate state can be acheived by the partial resistive switching of top PCM layer by repetative stimulation of smal voltage pulse.

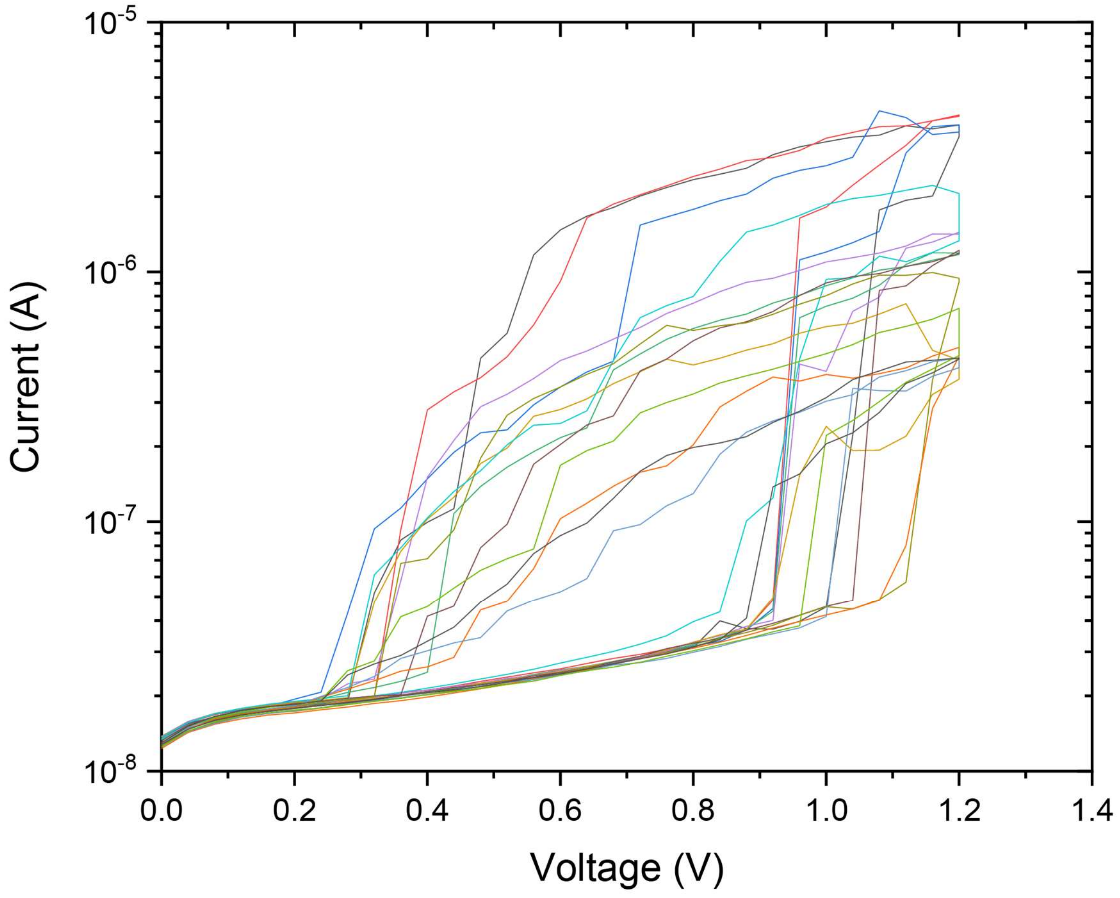


**Fig. S8 | Multilevel capability of TS-PCM cell**. The I-V curve shows the multilevel switching characteristics of TS-PCM. Using relatively harsh condition of voltage sweep (1.2 V), top PCM layer can be partially switched from amorphous to crystalline state. The gradual and nonvolatile resistive switching property of TS-PCM enables the emulation of long-term synaptic plasticity.

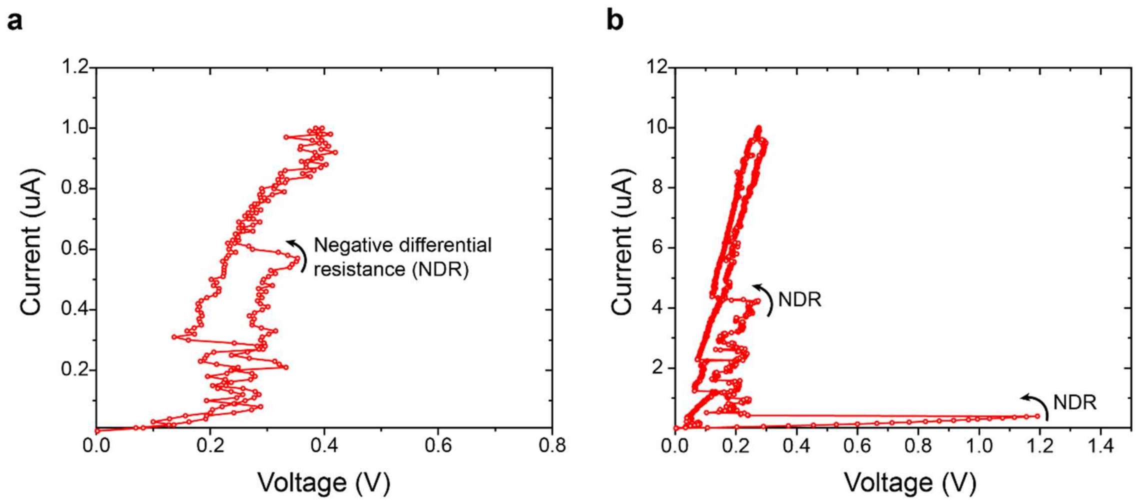


**Fig. S9 | Negative differential resistance of TS-PCM cell. a**, negative differential resistance (NDR) of TS-PCM in high resistance state (PCM OFF state). **b**, NDR of TS-PCM in low resistance state (PCM ON state). Both curves clearly exhibit the negative slope for current bias regime.

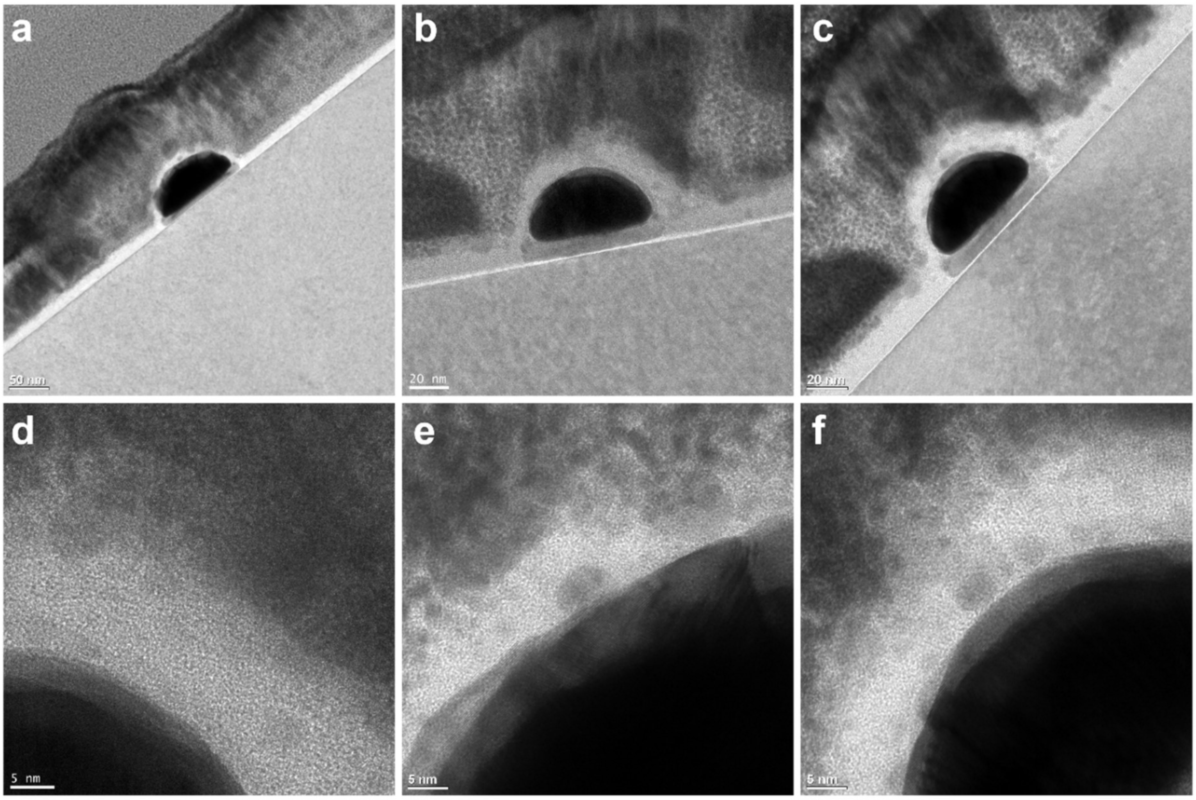


**Fig. S10 | Transmission electron misroscope (TEM) image of TS-PCM**. **a-c**, TEM images of TS-PCM in initial state, single pulse input, and multiple pulse input are shown in **a**, **b**, and **c**, respectively. **d-f**, magnified images of the Ag:SiO2 layer in **a**-**c**. Note that Ag clusters, the residues of voltaile Ag filament, increases with the number of applied voltage pulse cycles.

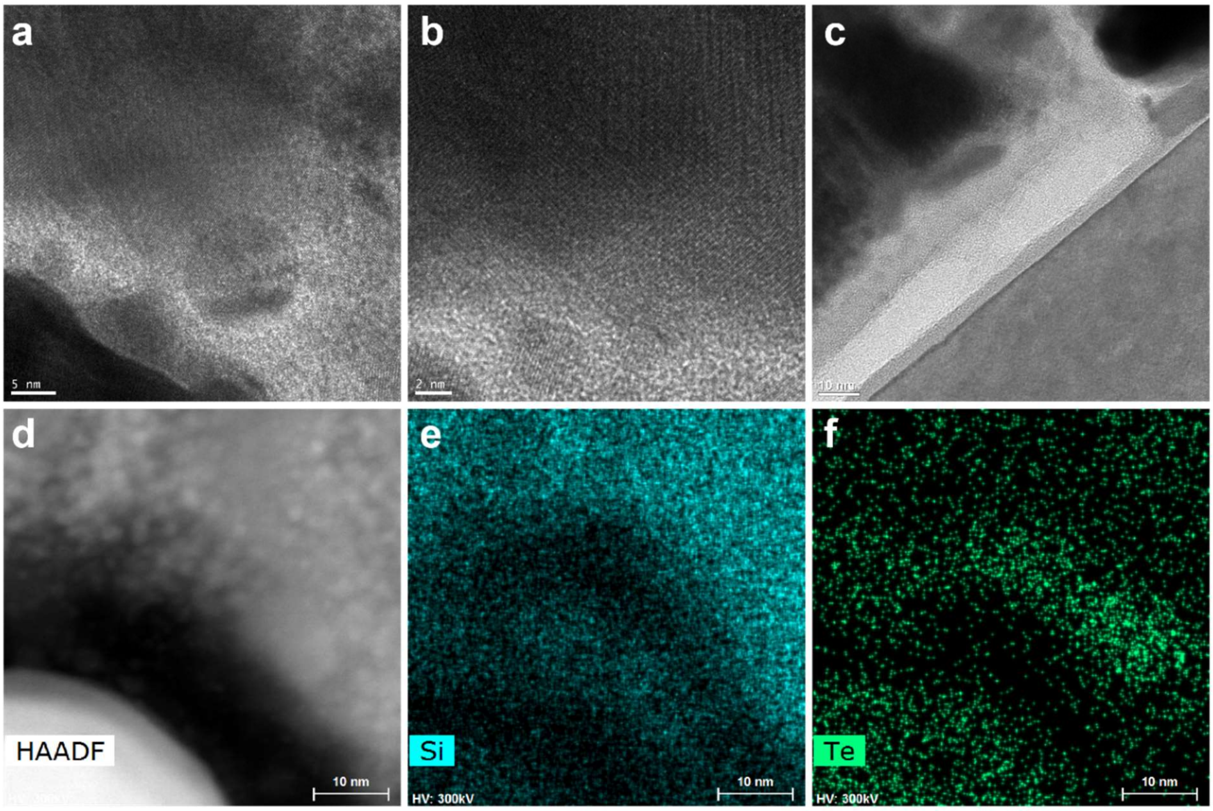


**Fig. S11 | TEM and Energy dispersive X-ray spectroscopy (EDS) image of TS-PCM**. **a**, Accumulation of Ag nanoclusters in GST/Ag:$SiO_2$ interface after degradation of the device by multiple application of 10 V pulse input. **b**, GST film in TS-PCM with low resistance state, which clearly shows the crystalline phase. **c**, TiW/GST/Ag:$SiO_2$/Ag layered structure located next to the bottom electrode. **d**, High-angle annular dark-field TEM image of TS-PCM. **e**-**f**, EDS images of Si and Te, which shows the layered structure of TS-PCM.

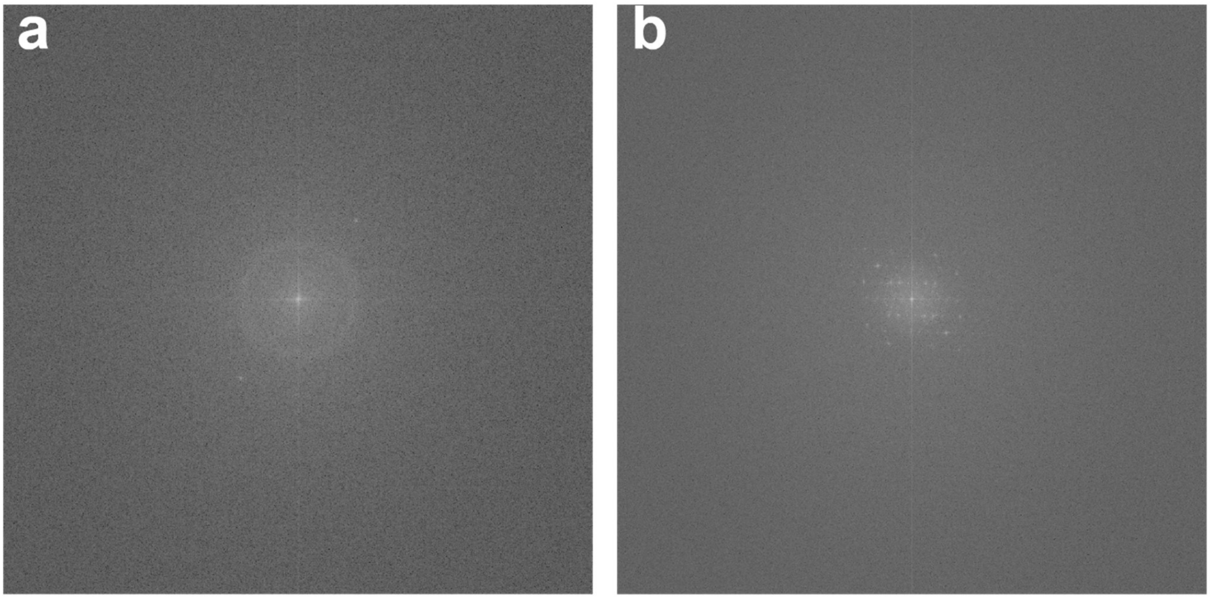


**Fig. S12 | Fast Fourier transform image of GST layer in TS-PCM**. **a**, FFT image of GST film from the supplementary Fig. S8d. **b**, FFT image of GST film from the supplementary Fig. S9b. The FFT image confirms the amorphous and crystalline phase of the GST layers.

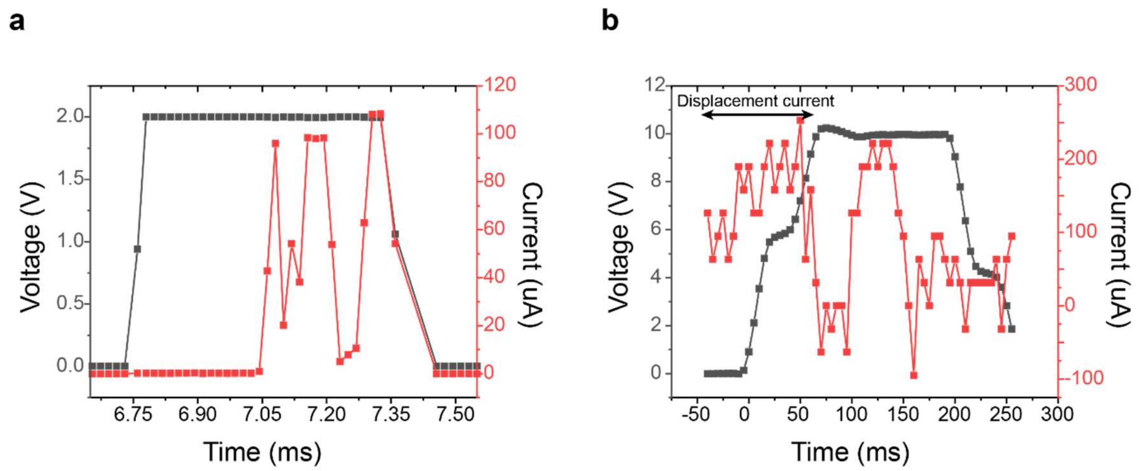


**Fig. S13 | Power consumption of TS-PCM cell. a**, set power consumptioin of TS-PCM cell. The set pulse with 2V amplitude and 600 μs width was applied resulting in the set power of 216 μW (129.6 nJ/bit). **b**, reset power consumption of TS-PCM cell. The reset pulse with 10 V amplitude and 150 ns width was applied resulting in the reset power of 2.21 mW (0.33 nJ/bit).

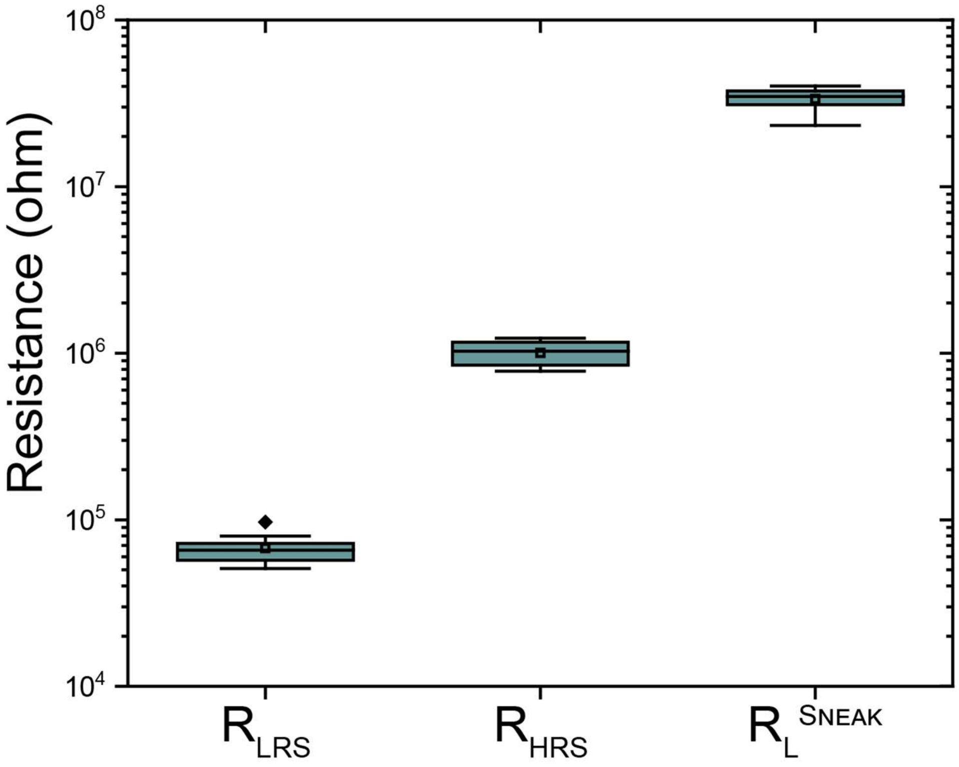


**Fig. S14 | Resistance errors of TS-PCM cell.** Due to the inherent noise in the resistive switching of TS-PCM, errors in resistance value are observed as shown in the figure. $R_{LRS}$, $R_{HRS}$, and $R_L^{sneak}$ are the LRS cell resistance, HRS cell resistance, and the resistance of unselected cell in LRS. These inherent errors affect to the readout error, decreasing the maximum crossbar array size of TS-PCM. See supplementary note 2 for array size calculation details. Center line, median; box limits, upper and lower quartiles; whiskers, 1.5x interquartile range; blank square, mean value; filled square, outliers.

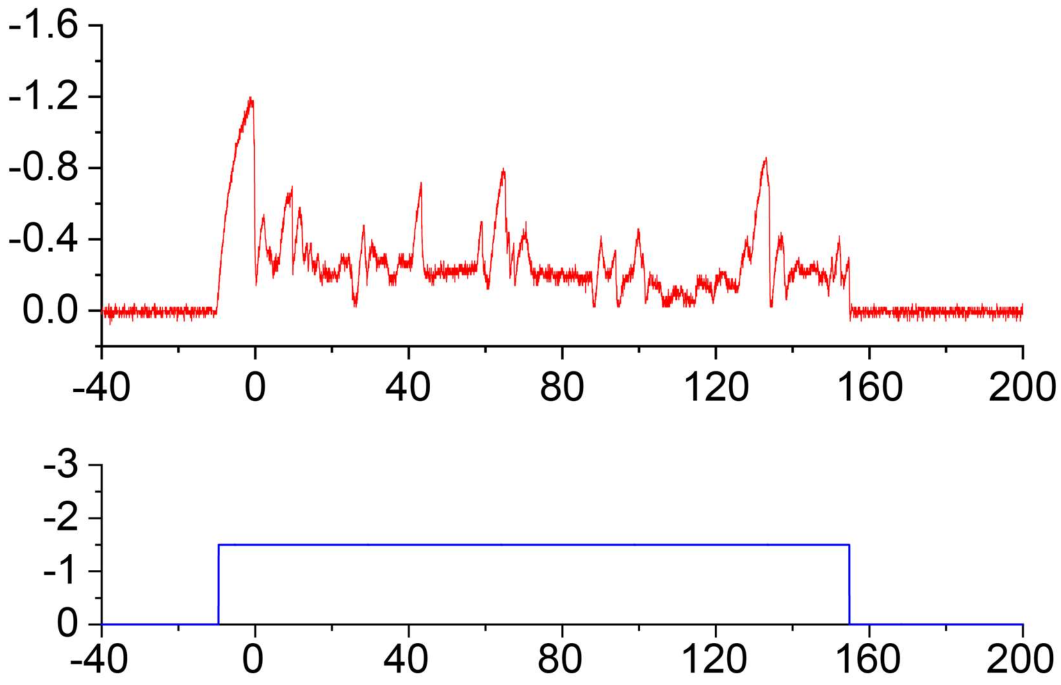


**Fig. S15 | Emulation of inhibition-induced spiking by TS-PCM RC circuit.** In addition to the tonic spiking and bursting, TS-PCM RC circuit can emulate the inhibition-induced spiking using negative current pulse input. An inhibition-induced spiking is observed in thalamo-cortical neurons, which fires action potential upon inhibitory current stimulation[3].

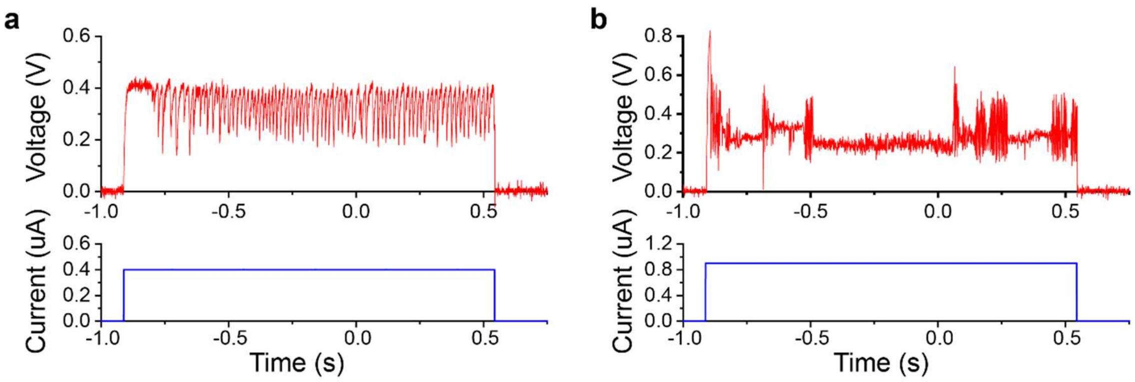


**Fig. S16 | Spiking emulation of TS-PCM without parallel capacitor. a**, tonic spiking behavoir of TS-PCM without parallel capacitor. b, tonic bursting behavior emulated using TS-PCM without parallel capacitor.

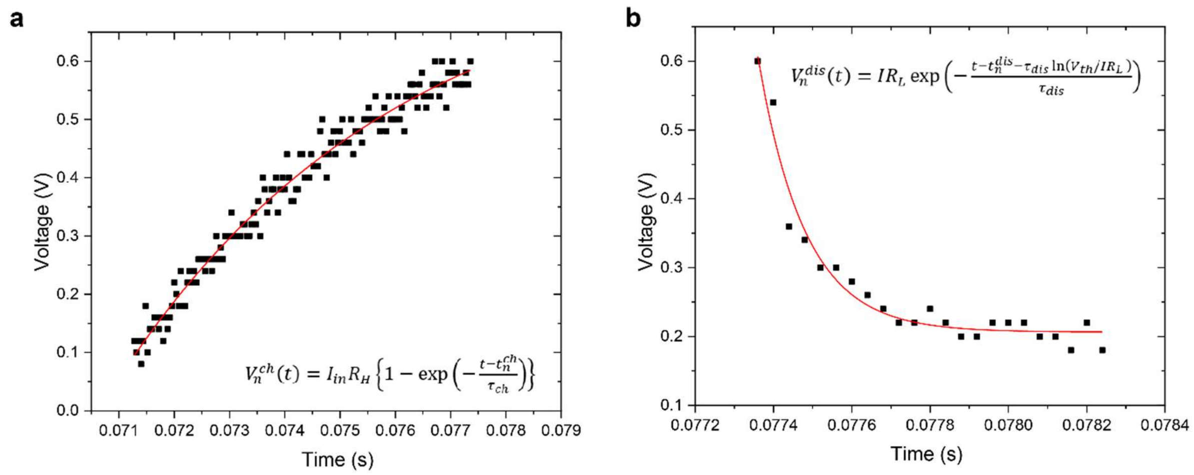


**Fig. S17 | Fitting of charging and discharging function of TS-PCM RC circuit**. **a-b**, Fitting result of spiking voltage charging equation and disch arging equation. The derivation of each equation is presented in supplementary note 2.

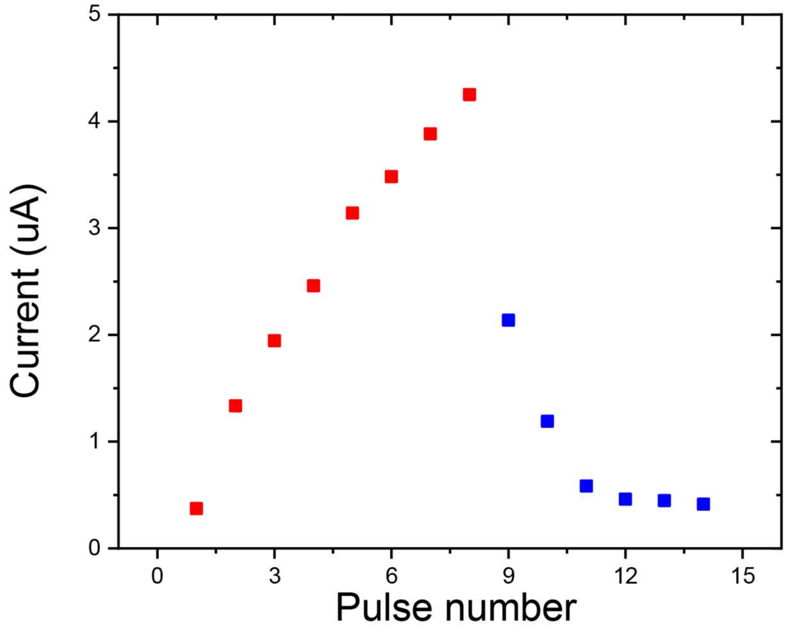


**Fig. S18 | Potentiation and depression characterisitic of TS-PCM cell.** Both potentiation and depression of synaptic weight is measured using consecutive pulse input. For potentiation process, 1.2 V/600 μs pulse was applied while 9 V/150 ns pulse was applied for the depression process.

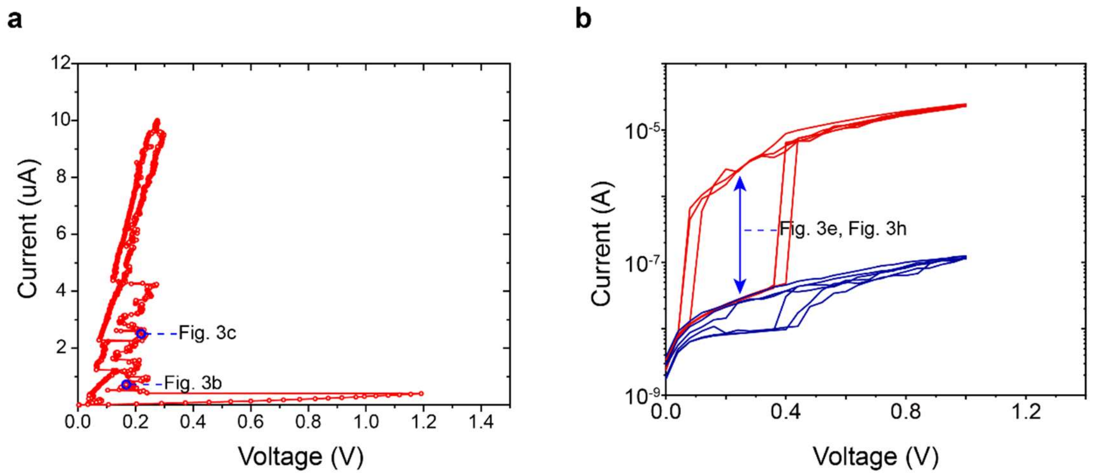


**Fig. S19 | Operation point of TS-PCM in Fig. 3. a**, operation point of Fig. 3b and Fig. 3c are shown in IV curve with current bias. **b**, operation point of Fig. 3e and Fig. 3h are shown in IV curve with voltage bias. Note that voltage values are different due to the pulse measurement. Generally, the threshold voltage of TS-PCM increases for smaller pulse width.

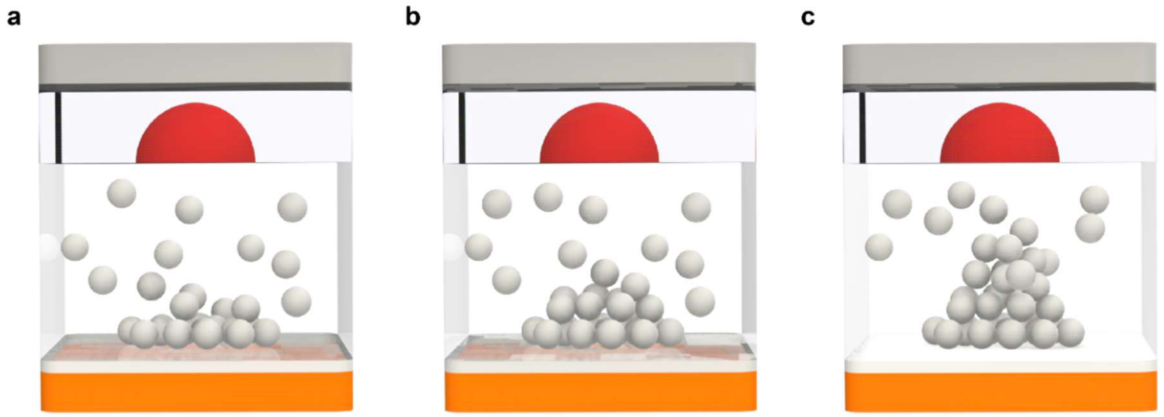


**Fig. S20 | Schematic illustration of residual Ag filament in bottom TS layer of TS-PCM.** **a**-**c**, The residual filament status with increasing input stimulation. At the initial state in **a**, only a small amount of Ag nanoclusters are present in the $SiO_2$ matrix. With increasing input cycles in **b** and **c**, filament residues significantly grows due to the lack of filament dissolution time. As a result, the threshold voltage of bottom TS layer decreases with consecutive input cycles. The decrease of threshold voltage is shown in the supplementary Fig. S16.

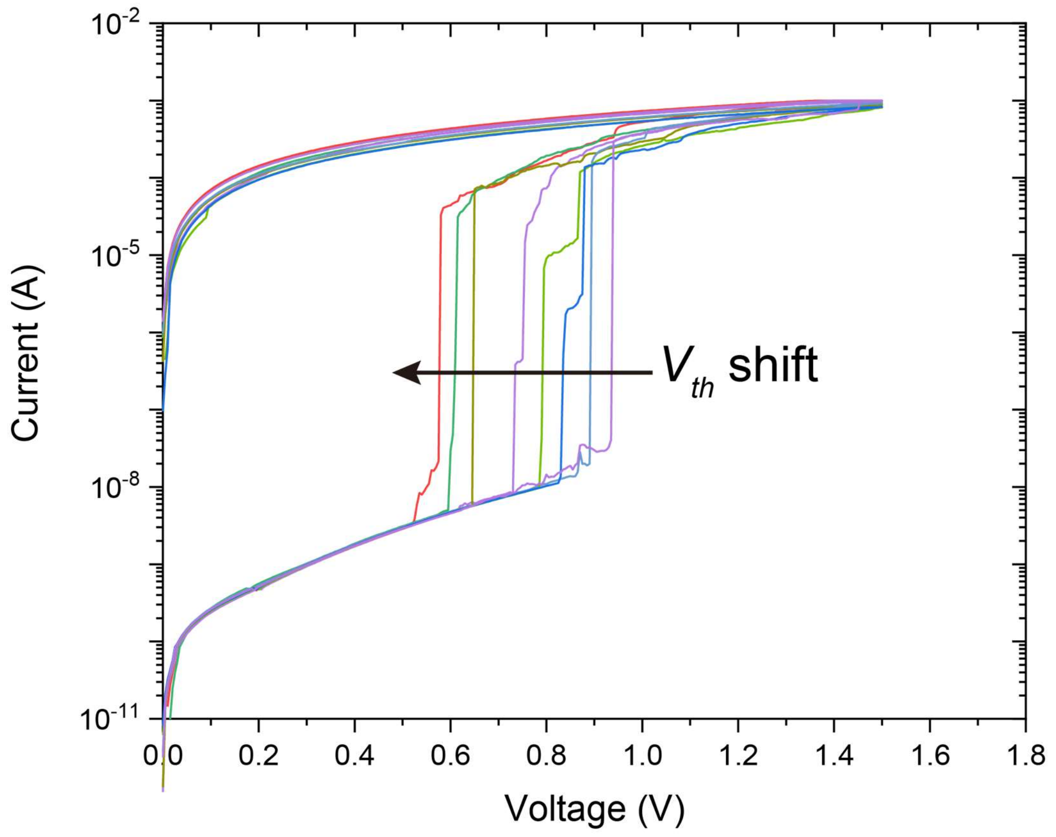


**Fig. S21 | The shift of threshold voltage in TS device.** To clearly demonstrate the threshold voltage shift behavior, consecutive voltage sweep is applied to TS device. the threshold voltage shows left-shift with increasing sweep cycles, confirming the growth of residual filament.

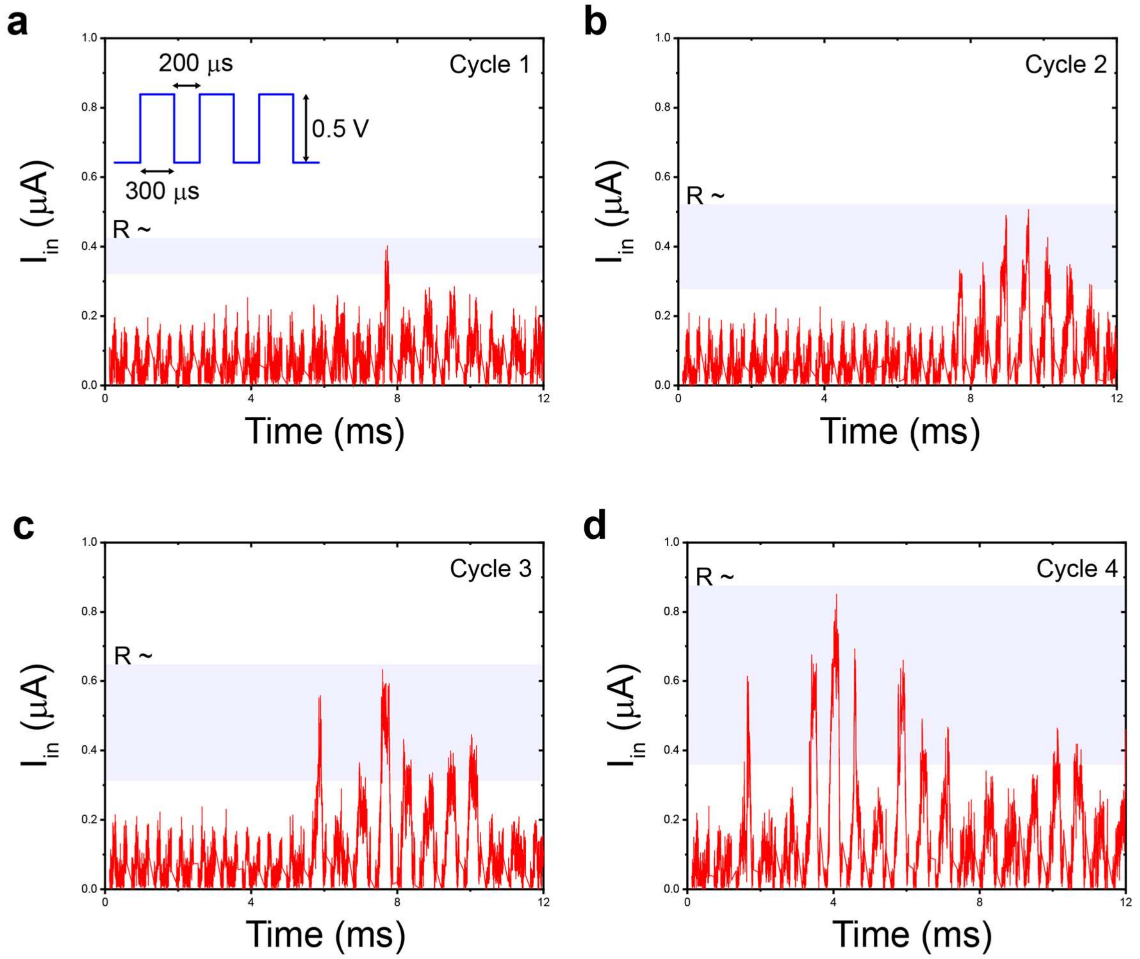


**Fig. S22 | Leaky-integrate and fire with intrinsic plasticity**. **a-d**, Emulation of the basic learning process using TS-PCM with LIF neuron model. Note that the spike number and spike amplitude increase simultaneously, which confirms the implementation of concomitant neuroplasticity and biological learning mechanism.

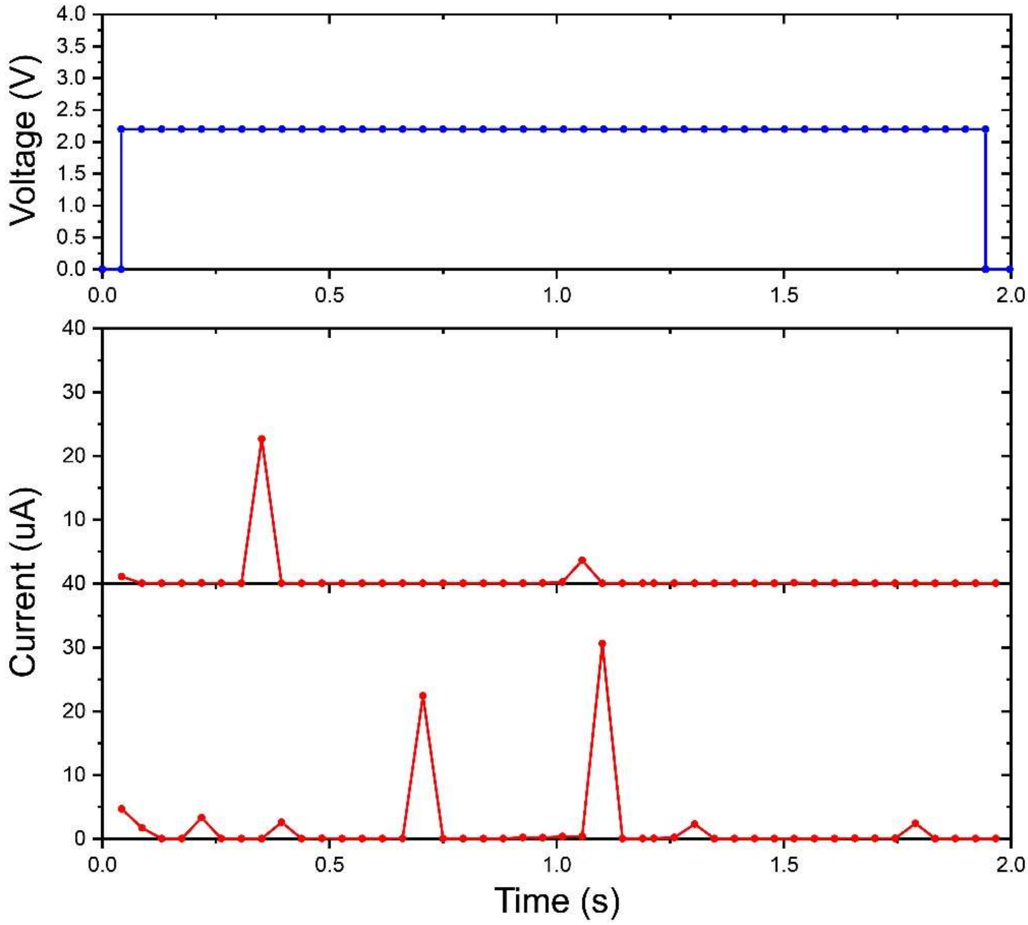


**Fig. S23 | Emulation of concomitant neuroplasticity with voltage bias using TS-PCM.** In addition to the current pulse input, voltage pulse can induce the concomitant neuroplasticity of TS-PCM. As shown in the upper panel, 2.2 V pulse input is applied to TS-PCM cell. As shown in the middel panel, only one voltage spike is observed in cycle 1. The lower panel demonstrates the out voltage of cycle 2, presenting two voltage spikes with higher output voltage. The simultaneous increase of spike number and voltage level indicates the implementation of concomitant neuroplasticity.

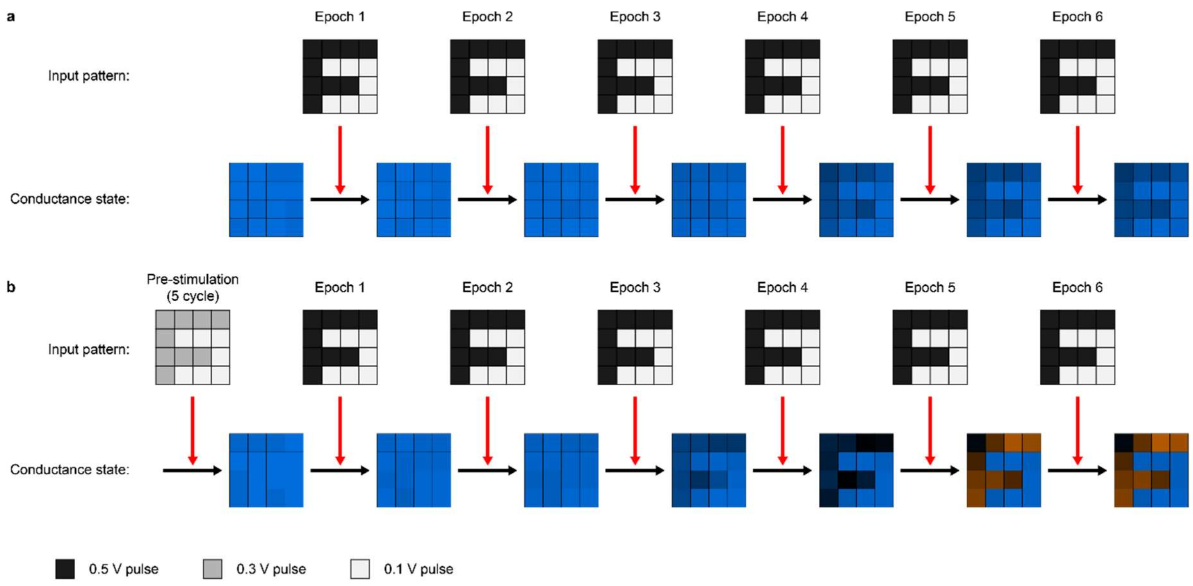


**Fig. S24 | Synaptic weight updates in crossbar structured TS-PCM array during naive training and feedback learning**. **a**, the evolution of conductance levels during the naive training shown in Fig. 5i. **b**, the evolution of conductance levels during the feedback training scheme. The resistance-based color map in Fig. 5i is reconstructed to clearly show the memorized pattern image. Compared to the naive training, the feedback learning shows higher acquistion rate, due to the hidden memory state.

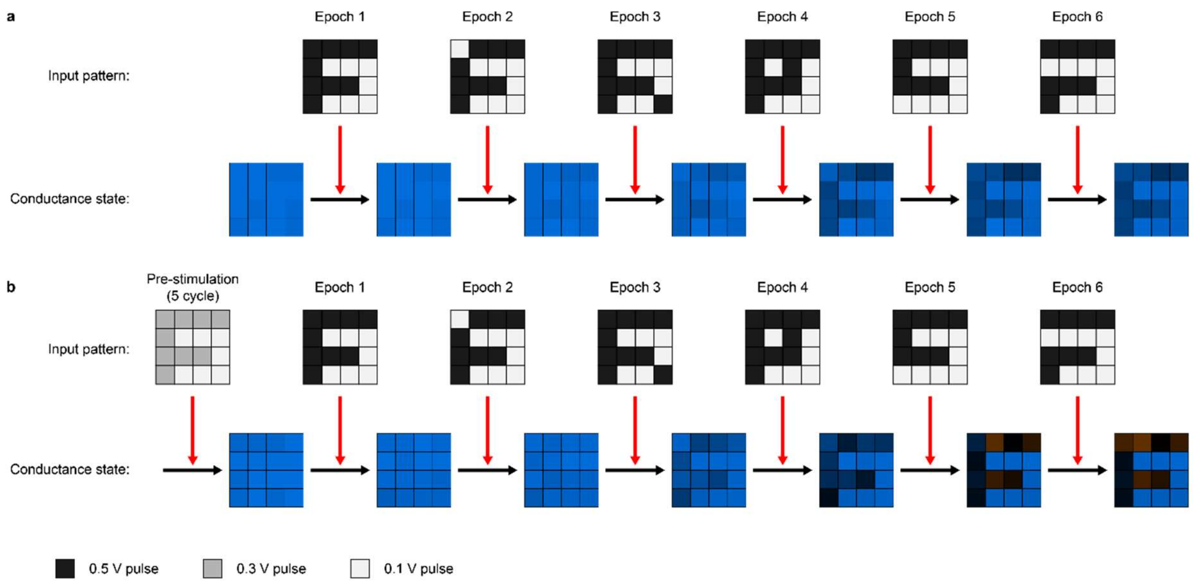


**Fig. S25 | Synaptic weight updates in crossbar structured TS-PCM array during naive training and feedback learning using the pattern with noise. a**, the evolution of conductance levels during the naive training. **b**, the evolution of conductance levels during the feedback training. Compared to the naive training, the feedback learning shows higher acquistion rate, due to the hidden memory state. Note that the enhacement of acquisition rate is lower than the clear pattern shown in supplementary Fig. S24.

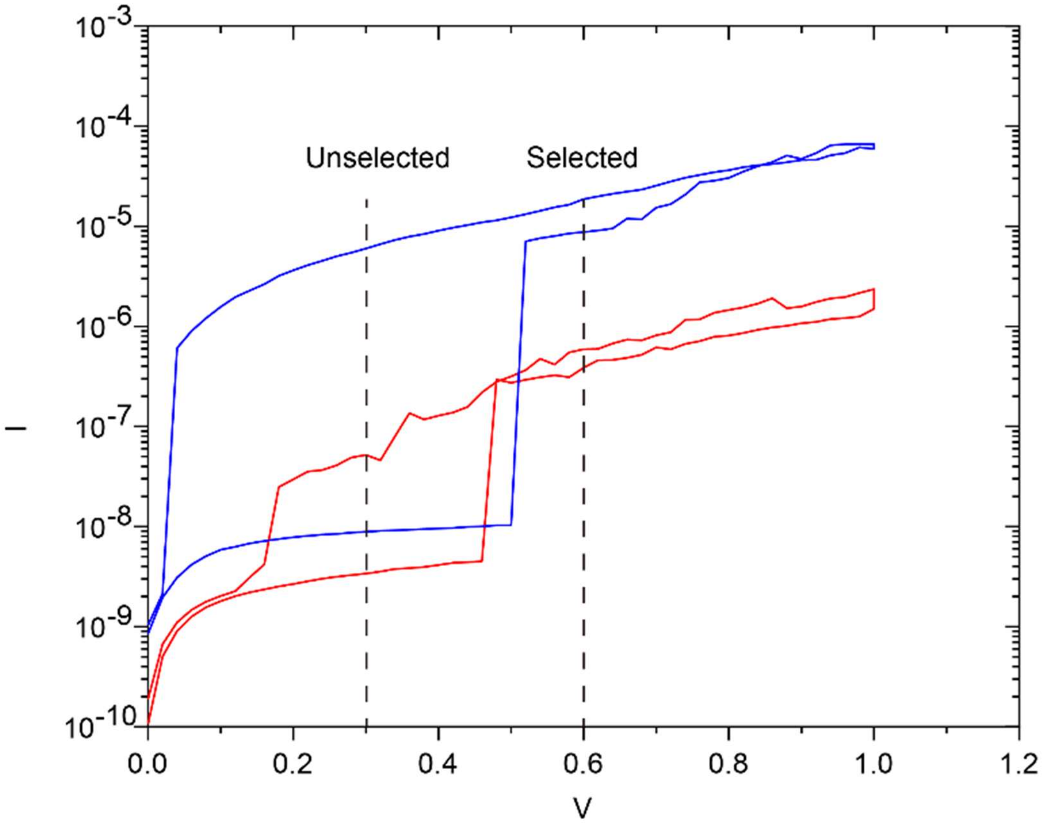


**Fig. S26 | IV curve of TS-PCM used for the calculation of readout margin in TS-PCM crossbar array**. Dotted lines indicate the applied voltage of the selected (0.6 V) and unselected cells (0.3 V), respectively.

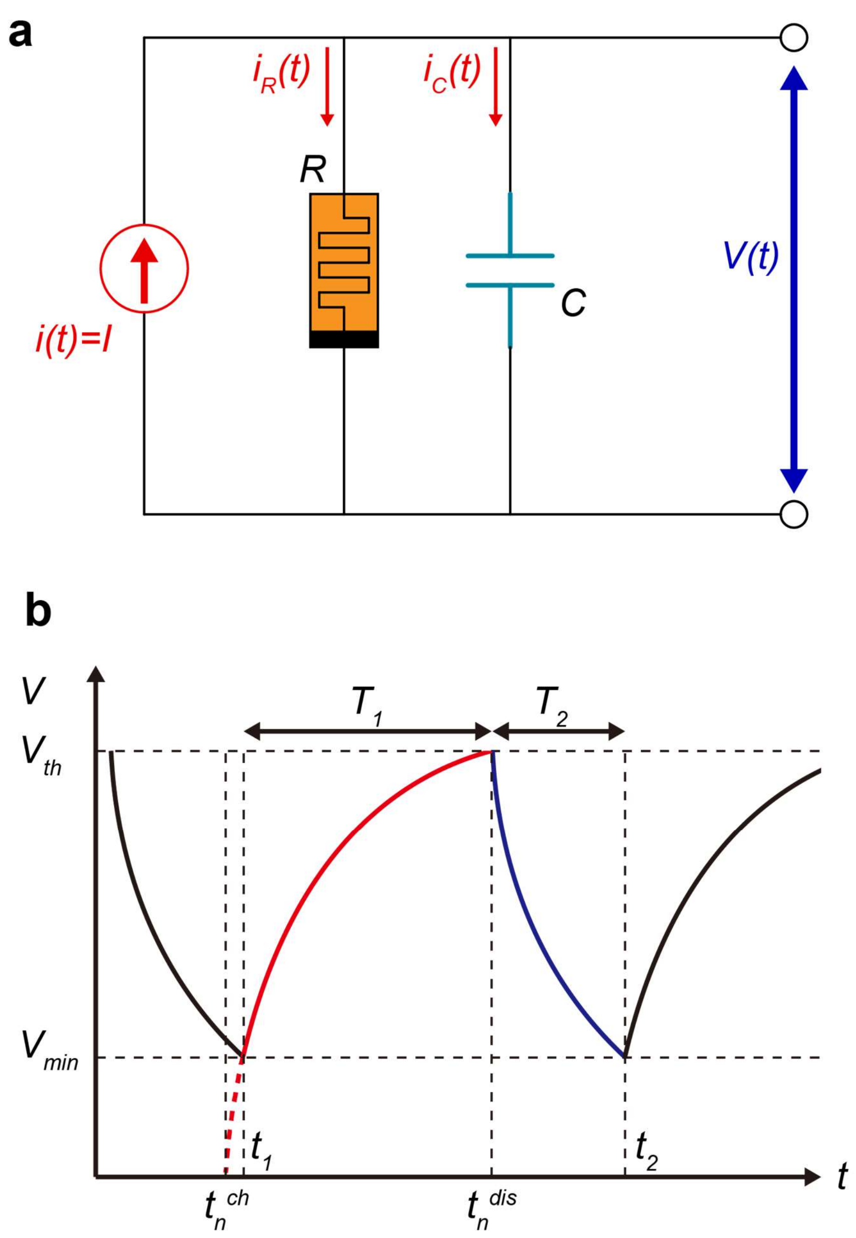


**Fig. S27 | RC circuit of TS-PCM with voltage spiking behavior**. **a**, Circuit representation of TS-PCM parallel RC circuit with constant current source $i(t)=I$. **b**, Schematic illustration of *n*th voltage spike in TS-PCM circuit. The voltage spiking behavior of TS-PCM circuit is presented in supplementary note 2.

**Supplementary note 1. Device model of the TS-PCM cell**

TS-PCM cell can be modeled by combining two compact models of phase change memory and threshold switch (diffusvie memristor). Ventrice *et al.* introduced a compact model of phase change memory as follows[1].

$$I_{PCM} = I_{\alpha}(V_1, V_{stat}) + F(I_{PCM}; I_{th}) \cdot [-I_{\alpha}(V_1, V_{stat}) + F(V_1;\ V_{hold}) \cdot I_{on}(V_1)] \quad (1)$$

where $V_{status}$ is a variable by input pulse shape, $I_{th}$ is the threshold current of the Ovonic Threshold Switching (OTS), and $V_{hold}$ is the holding voltage. The *F(x;a)* function is the Fermi-like smooth blending function modelling the switching of PCM resistance. $I_{\alpha}$ and $I_{on}$ describe the typical trend of chalcogenide-based resistors expressed as:

$$I_{\alpha}(V_1, V_{stat}) = \frac{1}{n_{stat} \cdot R_0(V_{stat}) \cdot \exp\left(\frac{E_a}{k} \cdot (\frac{1}{T} - \frac{1}{T_{ref}})\right)} [\exp(n_{stat} \cdot V_1) - 1] \quad (2)$$

with activation energy parameter $E_a$ and multiplication factor $n_{stat}$, low-field resistance $R_0$, Boltzmann constant *k*, and temperature *T*.

On the other hand, resitance of the TS device was modeled by Zhuo *et al.* using the filament diameter diameter *S* and the filament height *h*[2].

$$R_{TS}(h, S) = R_{on} \cdot \frac{h}{h_{max}} \cdot \sqrt{\frac{S_0}{S}} + R_{off} \cdot [\exp\left(\frac{h_{max} - h}{\lambda}\right) - 1]/[\exp\left(\frac{h_{max}}{\lambda}\right) - 1] \quad (3)$$

$$\frac{dh}{dt} = \beta V_2^n G(\mu_+, \sigma_+^2) - \gamma h G(\mu_-, \sigma_-^2)$$

$$\frac{dS}{dt} = \delta V_2^m \exp(-S) - \theta S$$

Here, $h_{max}$, $S_0$, and $\lambda$ are device constants determined by switching materials and cycling history. $\beta$, $n$, $\gamma$, $\delta$, and $\theta$ are electric field factor, the power of the electric filed fitting factor, diffusion fitting coefiicient for *h*, electric field fitting prefactor, and the diffusion fitting coeffcient for *S*, respectively. Note that $G(\mu, \sigma^2)$ is Gaussian function with fitting parameters.

For a programming voltage $V_{prog}$, voltage drop in PCM ($V_1$) and TS ($V_2$) layers are given as $V_{prog} = V_1 + V_2$. For the serial connection of PCM and TS devices, the current flowing through the devices are equal as $I_{PCM}(V_1) = I_{TS}(V_2)$. Using the ohm's law of $V = IR$:

$$V_2 = R_{TS}(h, S) \cdot I_{PCM}(V_1) \qquad (4)$$

Using the equations (1)~(4), compact model of the TS-PCM can be established, including both volatile and nonvolatile switching characteristics. Note that this compact model does not includes the Ag filament driven Joule heating effect, which may underestimate the intrinsic plasticity of the TS layer.

**Supplementary note 2. Calculation of readout margin in TS-PCM array by OBPU method**

For the worst case scenario, where all the unselected cells are in LRS and only the selected cell is in HRS, the circuit representation can be decribed as Fig. 2f. The normarlized readout margin of $N \times N$ crossbar TS-PCM array is given as follows[4-6]:

$$\frac{\Delta V}{V_{pull-}} = \frac{R_{pull-up}}{[R_{LRS} \| R_{Sneak}] + R_{pull-}} - \frac{R_{pull-up}}{[R_{HRS} \| R_{Sneak}] + R_{pull-up}} \qquad (1)$$

where $\Delta V$, $V_{pull\text{-}up}$, $R_{pull\text{-}up}$, $R_{LRS}$, $R_{HRS}$, and $R_{Sneak}$ are the readout voltage swing, pull-up voltage, pull-up resistance, LRS cell resistance, HRS cell resistance, and sneak path resistance, respectively. Note that the symbol $\|$ indicates the parallel resistance. Here, $\underline{R_{Sneak}}$ is sum of the resistance values in unselected cells:

$$R_{Sneak} = \frac{R_L^{Sneak}}{N-1} + \frac{R_L^{Sneak}}{(N-1)^2} + \frac{R_L^{Sneak}}{N-1} \qquad (2)$$

where $R_L^{sneak}$ is the resistance of unselected cell in LRS. By applying a $V_{read}$ of 0.6 V, 1/2 $V_{read}$ is applied in the resistance $R_L^{Sneak}/(N\text{-}1)$ and zero voltage is applied in $R_L^{Sneak}/(N\text{-}1)^2$ for sufficiently large $N$. From supplementary Fig. S26, the resistance values used in the calculation are $6.87 \times 10^4$ Ω, $1.01 \times 10^6$ Ω, and $3.38 \times 10^7$ Ω, for $R_{LRS}$, $R_{HRS}$, and $R_L^{Sneak}$, respectively. The resistance of the interconnecting electrodes is neglected for simplification. The calculation results in Fig. 2g indicate that the TS-PCM array can be scaled up to 316 × 316 for a 10 % readout margin, verifying the random-access capability in worst case scenario. In addition, the effect of noise on the maximum array size can be calculated using the resistance values with largest errors in supplementary Fig. S14. The resistance values for maximum noise case are $9.69 \times 10^4$ Ω, $7.78 \times 10^5$ Ω, and $2.32 \times 10^7$ Ω, for $R_{LRS}$, $R_{HRS}$, and $R_L^{Sneak}$, respectively. For the same readout margin of 10 %, possible array size of TS-PCM is calculated as 214 × 214 which

is far less than the average noise case.

**Supplementary note 3. Spiking voltage function in TS-PCM parallel RC circuit**

For a parallel RC circuit with current source *i(t)* shown in the supplementary Fig. S27a, the current *i(t)* can be expressed as follows:

$$i(t) = i_C(t) + i_R(t) = C\frac{dV(t)}{dt} + \frac{1}{R}V(t) \quad (1)$$

A partial differential equation is derived for a constant current source *i(t)=I* as shown in below equation:

$$\frac{dV(t)}{dt} = -\frac{1}{RC}V(t) + \frac{I}{C} \quad (2)$$

By solving this partial differential equation supposing that 0 V at $t_n^{ch}$ and $V_{th}$ at $t_n^{dis}$ for charging state and discharging states, respectively, the output voltage function is given as follows:

$$V(t) = A\exp\left(-\frac{t-TD_n}{RC}\right) + B \quad (3)$$

$$V_n^{ch}(t) = I_{in}R_H\left\{1 - \exp\left(-\frac{t-t_n^{ch}}{\tau_{ch}}\right)\right\} \quad (4)$$

$$V_n^{dis}(t) = IR_L\exp\left(-\frac{t-t_n^{dis}-\tau_{dis}\ln(V_{th}/IR_L)}{\tau_{dis}}\right) \quad (5)$$

where $TD_n$ is a timedomain of *n*th voltage spike. Note that the time constants of charging and discharging functions are different as $\tau_{ch} = R_HC$ and for $\tau_{dis}$, $= R_LC$, which can be estimated by fitting the equation (3) to the voltage spike data, as shown in the supplementary Fig. S17.

The period of voltage spike in TS-PCM RC circuit can be calculated from equation (4) and (5), where the period *T* of *n*th spike is defined as below:

$$T = T_1 + T_2 = \left(t_n^{dis} - t_1\right) + \left(t_2 - t_n^{dis}\right) \quad (6)$$

As shown in the supplementary Fig. S27b, charging period $T_1$ and discharging period $T_2$ can be calculated as follows:

$$V_n^{ch}(t_1) = V_{min} \ \Rightarrow \ I_{in}R_H\left\{1 - \exp\left(-\frac{t_1 - t_n^{ch}}{\tau_{ch}}\right)\right\} = V_{min}$$

$$\Rightarrow t_n^{ch} - t_1 = \tau_{ch}\ln(1 - \frac{V_{min}}{IR_H}) \quad (7)$$

$$V_n^{ch}(t_n^{dis}) = V_{th} \ \Rightarrow \ I_{in}R_H\left\{1 - \exp\left(-\frac{t_n^{dis} - t_n^{ch}}{\tau_{ch}}\right)\right\} = V_{th}$$

$$\Rightarrow t_n^{dis} - t_n^{ch} = -\tau_{ch}\ln(1 - \frac{V_{th}}{IR_H}) \quad (8)$$

$$V_n^{dis}(t_2) = V_{min} \ \Rightarrow \ IR_L\exp\left(-\frac{t_2 - t_n^{dis} - \tau_{dis}\ln(V_{th}/IR_L)}{\tau_{dis}}\right) = V_{min}$$

$$\Rightarrow t_2 - t_n^{dis} = \tau_{dis}\ln(\frac{V_{th}}{V_{min}}) \quad (9)$$

The sum of equation (7)-(9) defines the spike period $T$ as follows:

$$T = (t_n^{dis} - t_1) + (t_2 - t_n^{dis}) = (t_n^{ch} - t_1) + (t_n^{dis} - t_n^{ch}) + (t_2 - t_n^{dis}) \quad (10)$$

$$T = \tau_{ch}\ln\left(1 - \frac{V_{min}}{IR_H}\right) - \tau_{ch}\ln\left(1 - \frac{V_{th}}{IR_H}\right) + \tau_{dis}\ln\left(\frac{V_{min}}{V_{th}}\right)$$

$$= \tau_{ch}\ln\left(\frac{IR_H - V_{min}}{IR_H - V_{th}}\right) + \tau_{dis}\ln\left(\frac{V_{th}}{V_{min}}\right) \quad (11)$$

$$\therefore T = C[R_H\ln\left(\frac{IR_H - V_{min}}{IR_H - V_{th}}\right) + R_L\ln\left(\frac{V_{th}}{V_{min}}\right)] \quad (12)$$

From equation (12), the spike period $T$ shows clear dependency on the capacitance $C$, which was experimentally confirmed in Fig. 3d.

Supplementary References.


1. Ventrice, D., Fantini, P., Redaelli, A., Pirovano, A., Benvenuti, A., & Pellizzer, F. (2007). A Phase Change Memory Compact Model for Multilevel Applications. *IEEE Electron Device Letters*, 28(11), 973–975.

2. Zhuo, Y. et al. (2021). A Dynamical Compact Model of Diffusive and Drift Memristors for Neuromorphic Computing. *Advanced Electronic Materials*, 2100696 (2021).

3. Kumar A, Kansal S, Hanmandlu M. Classification of different neuron behavior by designing spiking neuron model. *2013 IEEE Int. Conf. Emerg. Trends Comput. Commun. Nanotechnol. (ICECCN)*; 2013: IEEE; 2013. p. 25-30.
4. Gül F. Addressing the sneak-path problem in crossbar RRAM devices using memristor-based one Schottky diode-one resistor array. *Results Phys.* **12**, 1091-1096 (2019).
5. Huang C-H, Chou T-S, Huang J-S, Lin S-M, Chueh Y-L. Self-Selecting Resistive Switching Scheme Using $TiO_2$ Nanorod Arrays. *Sci. Rep.* **7**(1), (2017).
6. You BK, Byun M, Kim S, Lee KJ. Self-Structured Conductive Filament Nanoheater for Chalcogenide Phase Transition. *ACS Nano* **9**(6), 6587-6594 (2015).
